# A natural experimental study of improvements along an urban canal: impact on canal usage, physical activity and other wellbeing behaviours

**DOI:** 10.1186/s12966-021-01088-w

**Published:** 2021-01-27

**Authors:** Jack S. Benton, Sarah Cotterill, Jamie Anderson, Vanessa G. Macintyre, Matthew Gittins, Matthew Dennis, David P. French

**Affiliations:** 1 Manchester Centre for Health Psychology, Division of Psychology & Mental Health, School of Health Sciences, University of Manchester, Coupland 1 Building, Oxford Road, Manchester, M13 9PL UK; 2 Centre for Biostatistics, Division of Population Health, Health Services Research & Primary Care, School of Health Sciences, University of Manchester, Manchester, UK; 3 Urban Institute, Department of Geography, School of Environment, Education and Development, University of Manchester, Manchester, UK; 4 Department of Geography, School of Education, Environment and Development, University of Manchester, Manchester, UK

**Keywords:** Physical activity, Wellbeing, Urban, Canal, Green space, Intervention, Natural experiment, Systematic observation

## Abstract

**Background:**

There are few robust natural experimental studies of improving urban green spaces on physical activity and wellbeing. The aim of this controlled natural experimental study was to examine the impact of green space improvements along an urban canal on canal usage, physical activity and two other wellbeing behaviours (social interactions and taking notice of the environment) among adults in Greater Manchester, UK. The intervention included resurfaced footpaths, removal of encroaching vegetation, improved entrances, new benches and signage.

**Methods:**

Two comparison sites were matched to the intervention site using a systematic five-step process, based on eight correlates of physical activity at the neighbourhood (e.g. population density) and site (e.g. lighting) levels. Outcomes were assessed using systematic observations at baseline, and 7, 12 and 24 months post-baseline. The primary outcome was the change in the number of people using the canal path from baseline to 12 months. Other outcomes were changes in physical activity levels (Sedentary, Walking, Vigorous), Connect and Take Notice behaviours. Data were analysed using multilevel mixed-effects negative binomial regression models, comparing outcomes in the intervention group with the matched comparison group, controlling for day, time of day and precipitation. A process evaluation assessed potential displacement of activity from a separate existing canal path using intercept surveys and observations.

**Results:**

The total number of people observed using the canal path at the intervention site increased more than the comparison group at 12 months post-baseline (IRR 2.10, 95% CI 1.79–2.48); there were similar observed increases at 7 and 24 months post-baseline. There was some evidence that the intervention brought about increases in walking and vigorous physical activity, social interactions, and people taking notice of the environment. The process evaluation suggested that there was some displacement of activity, but the intervention also encouraged existing users to use the canal more often.

**Conclusions:**

Urban canals are promising settings for interventions to encourage green space usage and potentially increase physical activity and other wellbeing behaviours. Interventions that improve access to green corridors along canals and provide separate routes for different types of physical activities may be particularly effective and warrant further research.

**Study protocol:**

Study protocol published in Open Science Framework in July 2018 before the first follow-up data collection finished (https://osf.io/zcm7v). Date of registration: 28 June 2018.

**Supplementary Information:**

The online version contains supplementary material available at 10.1186/s12966-021-01088-w.

## Background

There is mounting evidence that the use of urban green spaces can provide a range of mental and physical health benefits [1–3]. Urban green spaces are open spaces in urban areas wholly or partly covered by vegetation, ranging from ‘man-made’ spaces (e.g. parks) to more ‘natural’ spaces (e.g. woodlands), and can include landscapes around blue spaces (e.g. canals) [4]. Due to rapid global urbanisation, more people are living in dense built-up urban areas with limited access to high quality green space [5]. Therefore, improving or creating new urban green spaces is a potentially promising population-level intervention to promote health and wellbeing [6].

Numerous behavioural pathways have been proposed to explain how urban green spaces may promote health and wellbeing [7]. One of these behavioural pathways is physical activity, since many different physical activities can be performed in green spaces, including walking, running, cycling and sports; all of which have positive effects on physical and mental health [8, 9]. Physical activity undertaken in green spaces (‘green exercise’) may be more beneficial than physical activity done in other settings [10]. In addition to physical activity, urban green spaces can facilitate social interactions and cultivate social cohesion [11]; social relationships are critical for health and wellbeing [12]. Green spaces also offer opportunities for people to get outdoors and take notice of nature [13, 14], which can have important benefits for wellbeing [14, 15].

While there are multiple plausible behavioural pathways by which urban green spaces may influence health and wellbeing, the majority of research to date has focused on physical activity [16]. Growing cross-sectional evidence has found positive associations between accessibility, availability and quality of urban green spaces and physical activity [6]. However, cross-sectional evidence cannot determine causality and tells us little about how to design new or improve existing urban green spaces to increase physical activity. Due to researchers’ lack of control over environmental changes, making use of natural experiments is the optimal approach to understand the causal effects of environmental interventions [17]. Natural experiments are real world interventions that are not under the control of researchers. Researchers can design studies around a natural experiment to compare exposed and unexposed groups (or groups with different levels of exposure) to assess intervention effectiveness; known as natural experimental studies [18].

There is a dearth of natural experimental studies on the effect of urban green space interventions on physical activity and these studies have weak designs. A recent review of systematic reviews assessed the risk of bias in natural experimental studies of built environment interventions on physical activity [19]. From three existing exemplar systematic reviews, the researchers identified only 12 natural experimental studies (15 physical activity outcomes) with comparison groups, which included nine studies of urban green space interventions. All outcomes in these studies were assessed as having critical (*n* = 12) or serious (*n* = 3) risk of bias, thus showing that natural experimental studies conducted to date have questionable internal validity; a conclusion in line with other recent systematic reviews [16, 20–22].

Eight recommendations have been made to improve the methodological rigour of future natural experimental studies of environmental interventions [19], influenced by Medical Research Council (MRC) guidance for using natural experiments [18]. The present study implemented these recommendations: (1) publish study protocols with a priori analyses specified; (2) conduct sample size calculations; (3) better matching of comparison sites; (4) use multiple comparison sites; (5) use adequate outcome measurements; (6) measure intervention exposure at the individual level; (7) control for key confounders in statistical analyses; and (8) better reporting of samples and interventions.

Another limitation of the evidence base to date is that few natural experimental studies of urban green space interventions have been conducted in the United Kingdom (UK) and elsewhere in Europe; most have been in the United States (US) [17]. There are important differences between the UK and US that influence physical activity and make it hard to generalise findings, such as climate, residential density and population characteristics [23]. Also, most US studies are of park-based or large-scale redevelopment interventions [16]. As a result, the impact of small-scale replicable urban green space interventions, such as improving canals, greening residential streets or creating informal amenity green spaces, remains largely unknown.

Systematic observation (i.e. direct observations of behaviour using predetermined criteria) offers an objective method of unobtrusively assessing a range of behaviours that are important for wellbeing. The New Economics Foundation (NEF) conducted a review of the wellbeing literature on behalf of the UK Government’s Foresight project and identified five evidence-based behaviours that improve wellbeing (hereafter referred to as ‘wellbeing behaviours’), known as the ‘Five Ways to Wellbeing’ [24]: Be Active (engage in physical activity); Take Notice (awareness of one’s internal and external environment); Connect (socially interact with others); Keep Learning (acquire knowledge or skill in something new); and Give (pursue altruistic activities). The present study uses Method for Observing pHysical Activity and Wellbeing (MOHAWk) [25]: a recently validated observation tool which assesses three of these wellbeing behaviours (Be Active, Take Notice, Connect) in urban spaces.

## Research aim and objectives

The present study took advantage of an opportunity for a prospective natural experimental study. A private developer planned to implement new walking infrastructure and green space improvements along an urban canal in the UK (hereafter referred to as ‘canal improvements’). Canals are inland waterways that may produce important health and wellbeing benefits [26]; for example, areas surrounding canals can provide opportunities for a range of recreational activities, such as walking, jogging and cycling. To date, there are no natural experimental studies of the impact of interventions along urban canals on human health and wellbeing [27]. Growing investment into the regeneration of urban canals in the UK [28, 29] and elsewhere in Europe [30] increases the importance of producing robust practice-based evidence to inform decision makers how urban canals can effectively be used to improve population health and wellbeing.

The aim of this two-year natural experimental study was to evaluate the impact of the canal improvements on canal usage, physical activity and two other wellbeing behaviours (social interactions and taking notice of the environment) in adults. In line with the published study protocol [31], pre-planned objectives were to examine whether the canal improvements increased the following at 7, 12 and 24 months post-baseline, compared to two matched comparison sites where no changes occurred:

1. Total number of people (primary outcome at 12 months);
2. Walking and Vigorous physical activity behaviours (secondary outcomes);
3. Sedentary, Connect and Take Notice behaviours (exploratory outcomes).

An additional objective was to explore potential displacement of activity from a separate existing canal path, through process evaluation using intercept surveys and systematic observation.

## Methods

The study protocol is published elsewhere [31].

### Study design

This was a prospective controlled natural experimental study, with an intervention group (one intervention site) and a matched comparison group (two matched comparison sites). The study was set in Greater Manchester, UK: a large metropolitan authority containing 10 boroughs.

### Intervention

The intervention was implemented in the city of Salford, which is the 22nd most deprived local authority in England [32]. However, the intervention was located in an affluent area within Salford called Boothstown and Ellensbrook (population = 9532), which is the 2nd least deprived ward in Salford [33].

The intervention was implemented along the north side of an urban canal (Bridgewater Canal) and nearby connecting footpaths. There is an existing well-surfaced towpath on the south side of the canal, finished with a tarmac/ gravel surface, which has no vegetation other than a narrow grass strip between the path and the canal water. The intervention was designed, funded and implemented by Peel Land and Property Group (hereafter referred to as ‘Peel’): a private company which owns and manages 13 million square feet of property and 33,000 acres of land and water across the UK. According to Peel, the aim of the intervention was:

' … to enhance existing recreational provision which is used by the local community and the key connections between these recreational spaces. The improvements will enable and encourage circular walks to be undertaken by the local community in Boothstown. The improvements will help in creating pleasant routes for recreation and exercise to promote health and wellbeing, while also increasing biodiversity.' [34].

**Fig. 1:**
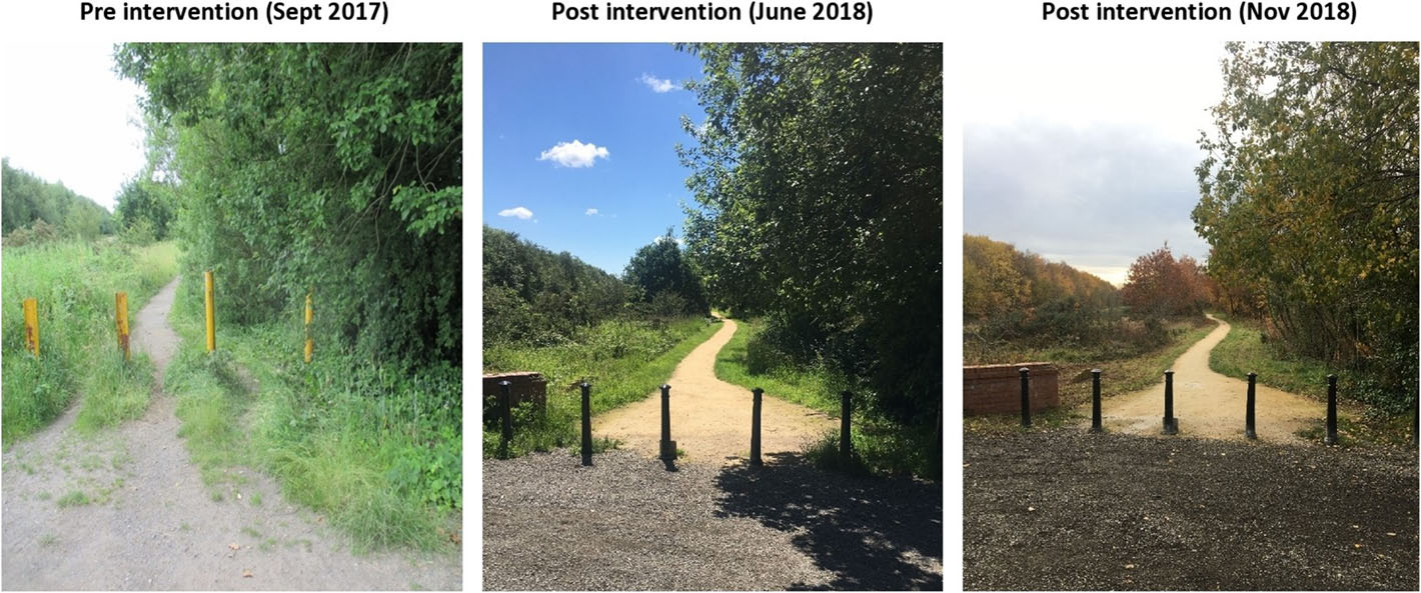
Intervention site pre and post intervention. *Photographs taken by Peel Land and Property Group and Jack Benton*

**Fig. 2:**
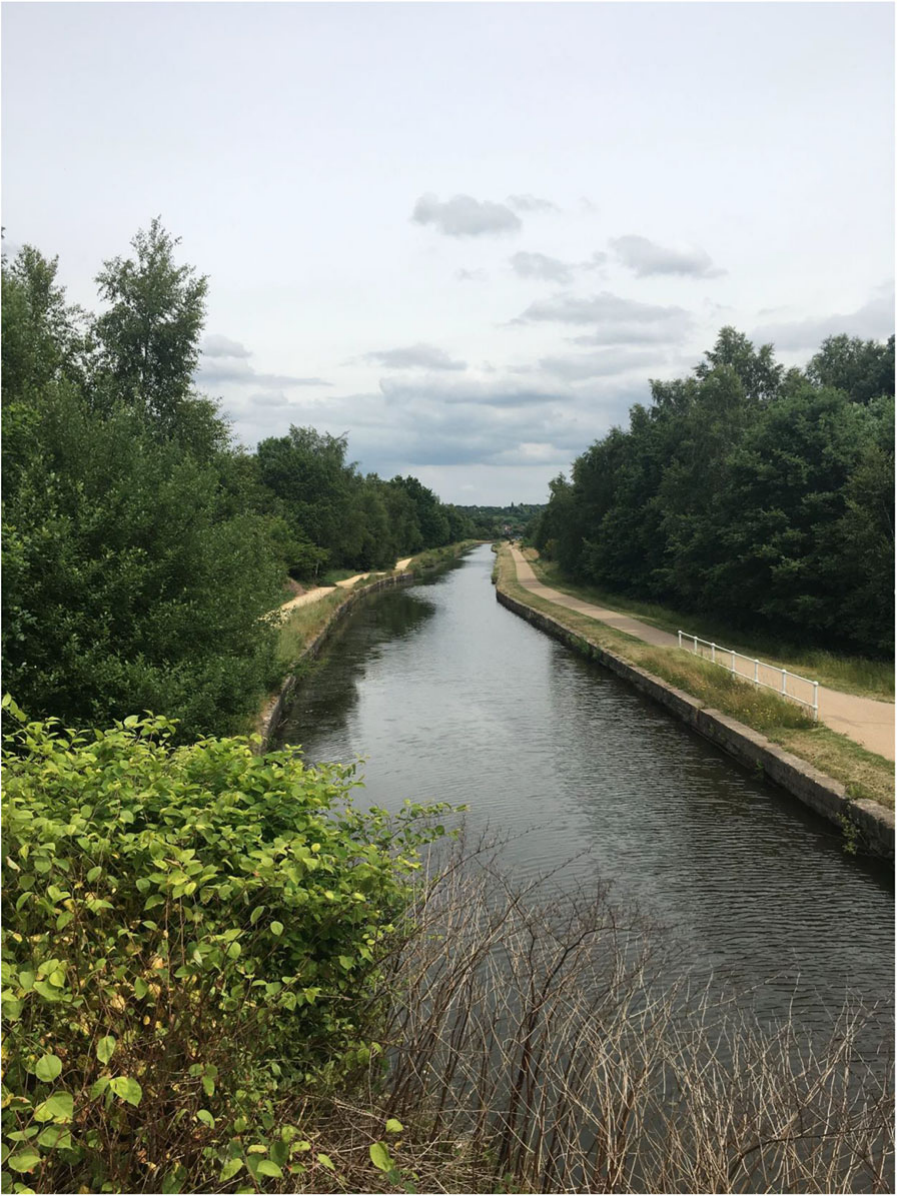
Intervention path (left side) and unchanged path (right side) post intervention. *Photograph taken by Jack Benton in June 2018*

There were two phases of intervention implementation. The main phase (Dec 2017 to May 2018) included new footpaths; resurfacing of existing footpaths using golden gravel; enhancement of an existing nature park and village green (e.g. improved entrance points); removal of encroaching vegetation; new benches, signage and informal play equipment. The second phase (Feb 2019) included new footpaths which link to a new nearby residential housing development due to be completed in 2021, although new residents started to occupy houses from early 2019. The total estimated cost of the intervention and 20-year ongoing maintenance is £920,000 (**≈** $1,242,000 US dollars), excluding Value Added Tax. Figure 1 shows the intervention site pre and post intervention. Figure 2 shows the intervention path on the north side of the canal and the existing unchanged path on the south side.

### Comparison group

**Fig. 3:**
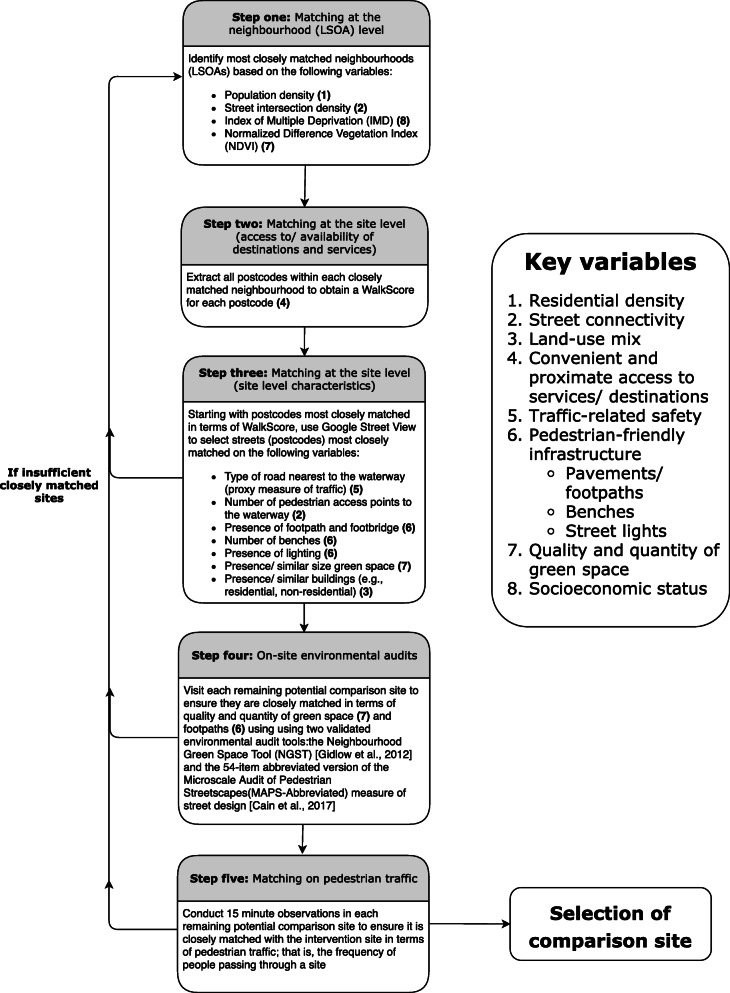
Overview of the five-step matching process used to identify comparison sites. *Numbers in brackets refer to the key variables used for matching. References for the environmental audit tools: Cain* et al. [35] *and Gidlow* et al. [36]*. Based on a similar graphic originally published in Benton* et al. [37]

We used a recently developed five-step process to identify two matched comparison sites in Greater Manchester (Fig. 3) [37]. The eight variables used for matching were based on nine systematic reviews of physical activity environmental correlates in adults [38–43] and older adults [44–46]. In brief, the first step involves identifying the most closely matched neighbourhoods to the index intervention neighbourhood, using spatial data at the Lower Layer Super Output Area (LSOA) level (population density, street connectivity, deprivation, neighbourhood greenness). The next four steps involve searching for the most closely matched comparison sites within the potential matched neighbourhoods identified in step one, using variables at the site level (e.g. footpath, benches, lighting). Steps two and three are conducted using Google Street View to narrow down potential matched comparison sites. Steps four and five involve in-person site audits. Additional file 1 provides further details of this matching process.

The two matched comparison sites were pooled together into one comparison group. The main reason for pooling the comparison sites was to increase statistical power. Also, including multiple comparison sites in the analysis provides increased confidence that any variation in confounding variables across comparison sites is offset, therefore reducing the risk that the intervention effect is confounded by site-specific variables in a single comparison site.

**Fig. 4:**
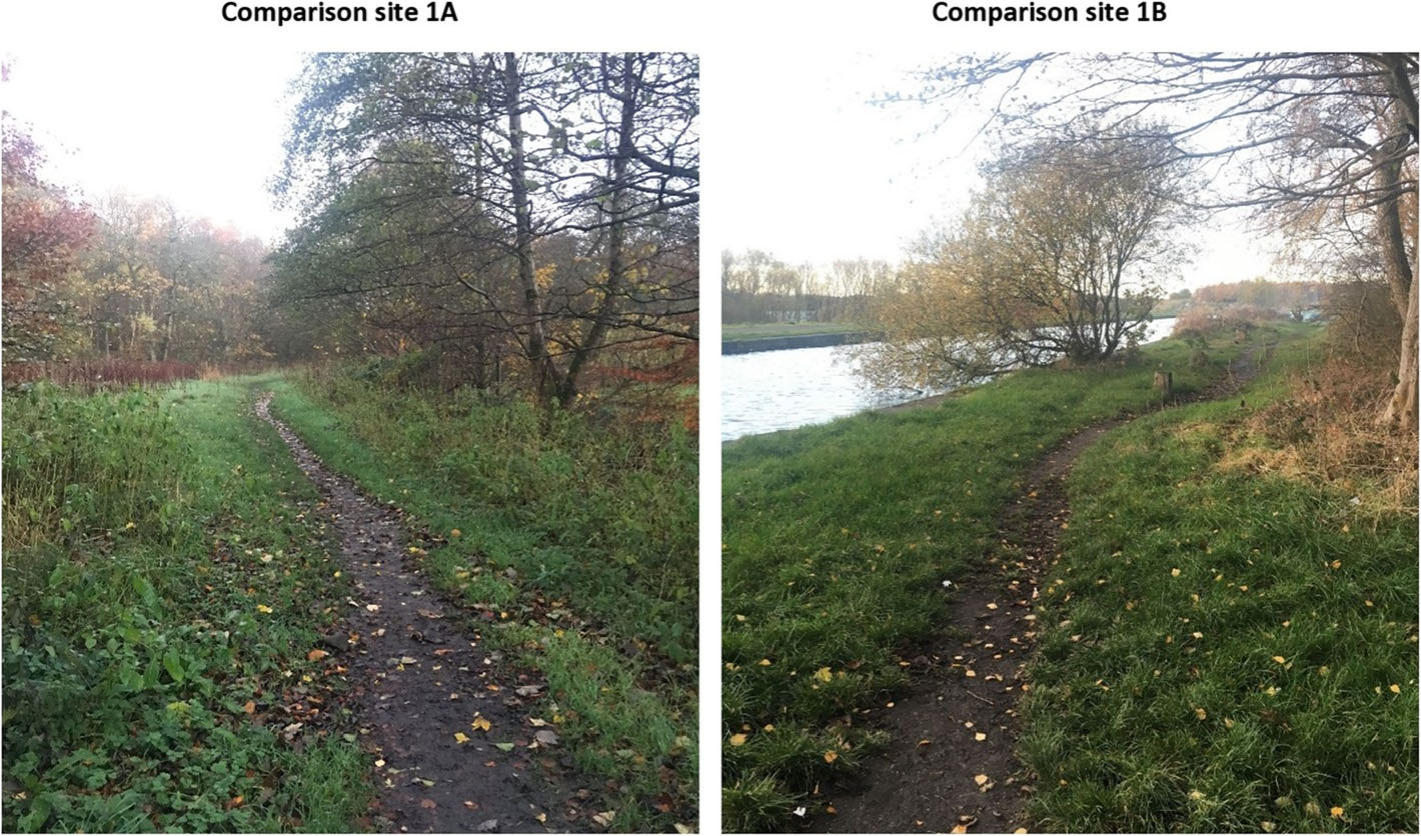
Comparison sites at baseline. *Photographs taken by Jack Benton in Nov 2017*

A description of the key characteristics of the intervention and two comparison sites can be found in Table 1. Figure 4 shows the comparison sites at baseline.

**Table 1:** Key characteristics of all study sites and LSOAs at baseline

Site	Description of sites at baseline	Ward (postcode)	WalkScore ^a^	LSOA ^b^ characteristics			
				Population density ^c^	Intersection density ^d^	IMD ^e^	NDVI ^f^
Intervention site	Canal with paths on both sides (one unsurfaced and one surfaced); two access routes; a bridge; no benches; no lighting; links to Bridgewater Nature Park and Vicars Hill Village Green	Boothstown and Ellenbrook (M28 1JD)	35	22.67	9.18	4.28	0.55
Comparison site 1A	Brook with paths on both sides (both unsurfaced); two access routes; a footbridge; no benches; no lighting; links to Jumbles Country Park	Bromley Cross (BL2 3EQ)	48	23.91	7.58	8.37	0.54
Comparison site 1B	Canal with two paths on both sides (one unsurfaced and one surfaced); two access routes; a footbridge; no benches; no lighting; links to Pennington Flash Country Park	Leigh West (WN7 4QP)	50	37.93	9.68	27.05	0.52

^a^ WalkScore uses a Google search algorithm to calculate a weighted score (1–100) based on the number and accessibility of amenities (such as shops and parks) within a 1-mile radius of a user-entered postcode, whereby closer amenities with the most accessible walking routes are weighted more strongly, used as a measure of ‘access to/ availability of destinations and services’. Higher scores indicate more ‘walkable’ areas

^b^ Lower Layer Super Output Area (LSOA): census reporting units containing between 1000 and 3000 people

^c^ Population density: number of persons per hectare; used as a proxy measure of residential density. Higher values indicate areas with higher population density

^d^ Intersection density: the number of 3-way junctions per 1000mˉ^2^ standardised by LSOA area; used as a measure of street connectivity. Higher values indicate areas with higher street connectivity

^e^ Index of Multiple Deprivation Score (IMD) [47]: an area deprivation score that combines multiple indicators of deprivation including income, employment, health and crime. Higher values indicate more deprived areas

^f^ Normalised Difference Vegetation Index (NDVI): a validated normalised scale of healthy vegetation cover; used as a measure for presence of greenery at the neighbourhood-level. Higher values indicate areas with more healthy vegetation cover

### Study sample

Data were collected on adults observed using the intervention and comparison sites.

### Outcomes

**Fig. 5:**
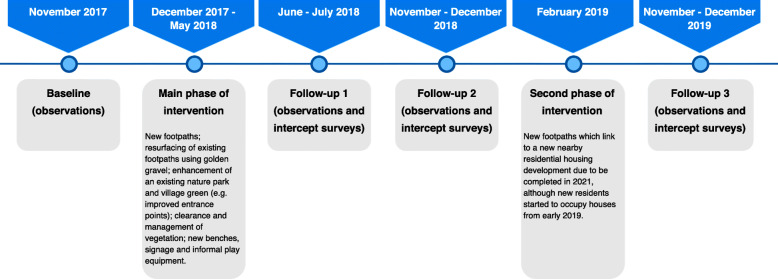
Timeline of data collection and intervention implementation

Systematic observations were conducted at baseline (Nov 2017) and three separate follow-ups: 7 months (June 2018); 12 months (Nov 2018); and 24 months (Nov 2019) post-baseline. Observations were conducted during 4 hour-long observation periods (8-9 am, 10-11 am, 1-2 pm, 3-4 pm) on 3 days (Tuesday, Wednesday, Saturday) at each site i.e. 12 hours of observations for each site at each time point. However, at 24 months, two observation periods at the intervention site were cancelled because of safety concerns for the observer, which involved the observer being approached in a hostile manner by a member of the public. In line with the procedure specified in our approved ethics application, the researcher left the area immediately and we ceased all remaining observations at that site. This incident was reported to the institutional Research Ethics Committee; no further action was required. Figure 5 provides an overview of data collection and intervention timings.

Observations were conducted using MOHAWk (Method for Observing pHysical Activity and Wellbeing): a systematic observation tool for assessing three levels of physical activity (Sedentary, Walking, Vigorous) and two other wellbeing behaviours (Take Notice: taking notice of the environment; Connect: social interactions) in urban spaces [25]. To date, there are over 650 h of MOHAWk data across 36 unique sites in Greater Manchester, Belfast and Valencia. There is now evidence of high inter-rater reliability between pairs of observers when using MOHAWk and criterion-related validity [25]. MOHAWk has been used in two separate natural experimental studies of urban green space interventions [48, 49].

Two observers were used at each time point, with each observer independently collecting data at one site. As there were three study sites but only two observers, observations were counterbalanced to control for week, day of the week, and time of day. The same observation schedule was used at all time points; this observation schedule provides a reliable estimation of activity [25, 50]. Observations were carried out regardless of weather conditions; observation periods were coded as high precipitation if the observer recorded an accumulated duration of any precipitation lasting for 50% or more of the observation period (i.e. 30 min or more), as recommended by MOHAWk procedures.

The target area at the intervention site was the path identified for improvements (‘intervention path’). Observers recorded anyone who entered onto the intervention path, regardless of which direction they were coming from. Target areas at the two comparison sites were defined to resemble the target area at the intervention site, by capturing people who entered the unsurfaced canal side path regardless of which direction they were coming from.

At each time point, JB (first observer) trained the second observer using the MOHAWk instruction manual and by practicing observations in the study sites. At the end of training, inter-rater reliability was assessed between the pair of observers for 2 hours using 5-min observation periods (i.e. 24 data points). Inter-rater reliability for counts of people, their characteristics and behaviours was ‘good’ or ‘excellent’, analysed using two-way mixed, single measure, consistency intraclass correlation coefficients (ICCs) (< 0.5 = poor; 0.5–0.75 = moderate; 0.76–0.9 = good; and > 0.9 = excellent [51]).

### Logic model

Hypothesised causal pathways were outlined in the logic model (see study protocol [31]), based on a framework suggested by Panter et al. [52]. The model predicted that the intervention would improve accessibility, convenience and pleasantness of the canal route and key destinations, which would in turn encourage more people to use the canal. This, in turn, would lead to observable increases in physical activity, social interactions, and people taking notice of the environment.

### Sample size

We used the method suggested by Donner and Klar ([53], p.66) for calculating the sample size for a matched pair design: calculate the number of clusters (sites) required for a completely randomised cluster design, and then multiply that by one minus the correlation between the mean outcomes in the two groups. This was conducted for the primary outcome measure: the total number of people per hour (observation period). Due to the absence of studies of urban canal interventions, the sample size calculation was informed by MOHAWk data from a feasibility study of two UK residential streets [37]. Based on these data, we assumed a mean number of people per hour in the comparison group of 20, a standard deviation in both groups of 27, and a correlation between intervention and comparison sites of 0.37. We assumed an intracluster correlation coefficient of 0.02, meaning that 2% of the variation in any particular outcome can be explained by variation between clusters (sites): this relatively low value has been used in previous studies that have evaluated small urban spaces (B. Han, personal communication). We calculated that matching one intervention site (intervention group) and two comparison sites (comparison group), with 12 observation periods per site, provided 80% power (*p* = .05, two-sided test) to detect an increase in the mean number of people from 20 counts per hour in the comparison group to 80 counts per hour in the intervention group. The calculation was conducted using the *clsampsi* command in Stata version 14.1.20.

### Analyses

The unit of analysis was at the level of the observation period i.e. counts per observation period per site. Analyses were conducted separately for data at 7, 12 and 24 month follow-ups, in order to produce separate effect estimates for each follow-up time point. We originally planned to analyse adults and older adults separately, but combined them to increase statistical power. Analyses were conducted using Stata version 14.1.20.

#### Primary outcome – total number of people using the canal

We estimated the effect of the intervention on the total number of people counted per observation period, compared to the comparison group, controlling for three covariates: day (Tuesday, Wednesday, Saturday), time of day (8-9 am, 10-11 am, 1-2 pm, 3-4 pm) and precipitation (Yes or No). Precipitation was a binary variable, which was coded as ‘Yes’ if the observer recorded an accumulated duration of any precipitation lasting for 50% or more of the observation period (i.e. 30 min or more).

Multilevel mixed-effects negative binomial regression models were used because they can account for overdispersion [54]. We specified a random intercept at the site level to take into account any unobserved differences between sites. We used robust standard errors to obtain unbiased standard errors. Results were reported as incidence rate ratios (IRRs), which represent the relative change in the total number of people at the intervention group compared to the comparison group.

We followed three steps to build a suitable negative binomial regression model. First, using baseline data only (without group allocation), we built a regression model to examine the relationship between the baseline data and covariates. Second, we combined the baseline and follow-up data and applied the same regression model from step one. Third, we added the Intervention Group (intervention or comparison) and Time Point (baseline or follow-up) into the model. The intervention effect was the interaction of the Intervention Group and Time Point. This is a difference-in-differences approach i.e. analysis of the change in outcomes between intervention and comparison groups pre- and post-intervention, using a regression with terms for group, period, and the group-by-period interaction [55]. Model estimates were obtained using the *menbreg* Stata command.

#### Secondary and exploratory outcomes – physical activity and other wellbeing behaviours

For some of the physical activity levels (Sedentary, Walking, Vigorous) and other wellbeing behaviours (Connect, Take Notice), the number of observations was too low to undertake statistical modelling as originally planned. Instead, we calculated the change in counts of each behaviour from baseline to follow-up for each of the intervention and comparison groups. Mann-Whitney U tests were then used to assess whether any differences in the change in counts between intervention and comparison groups were statistically significant, at a priori alpha level of 0.05. There were no adjustments for multiple comparisons.

#### Sensitivity analyses

To assess for any potential bias due to precipitation when analysing the physical activity and other wellbeing behaviour outcomes, observation periods were removed if precipitation occurred for 50% or more of the observation period (recorded by the observer).

### Process evaluation

#### Systematic observation

At the intervention site, observers separately recorded people who accessed the existing unchanged path on the other side of the canal (Fig. 2). Specifically, these were people who were following the same route that leads to the intervention path, but did not use the intervention path and instead travelled over the bridge to use the unchanged path on the other side of the canal. This allowed us to assess the extent to which the intervention affected use of the unchanged path.

#### Intercept surveys

Short face-to-face intercept surveys were conducted to assess change in use of the intervention path and potential displacement of activity from the unchanged canal path at the intervention site. Surveys were conducted at each of the follow-ups (June/ July 2018; Nov/ Dec 2018; Nov/ Dec 2019) using the same schedule as observations but on different days. Surveys were attempted with all English-speaking adult canal users (aged 18+ checked in introduction). Informed consent was obtained verbally and surveys were completely anonymous. The estimated gender, age group and ethnicity of each participant were recorded using MOHAWk coding procedures. Additional file 2 contains the main intercept survey questions.

## Results

Table 2 provides information on baseline observation periods, sample and outcomes. The intervention and comparison group samples were similar at baseline in terms of gender and ethnicity. Although the intervention site had higher numbers of people at baseline, proportions of wellbeing behaviours were similar between intervention and comparison sites.

**Table 2:** Baseline information on observation periods, sample and outcomes

	Comparison group (2 sites)	Intervention group (1 site)
**Observation periods**		
Total number of observation periods	24	12
8 am – n (% of total)	6 (25%)	3 (25%)
10 am – n (% of total)	6 (25%)	3 (25%)
1 pm – n (% of total)	6 (25%)	3 (25%)
3 pm – n (% of total)	6 (25%)	3 (25%)
Tuesday – n (% of total)	8 (33.33%)	4 (33.33%)
Wednesday – n (% of total)	8 (33.33%)	4 (33.33%)
Saturday – n (% of total)	8 (33.33%)	4 (33.33%)
High precipitation – n (% of total)	5 (20.83%)	0 (0%)
**Sample**		
Female – n (% of total)	31 (47.69%)	30 (58.82%)
White – n (% of total)	65 (100%)	50 (98.04%)
**Primary outcome**		
Overall count of people – n (median, IQR)	65 (2, 1.75)	51 (4, 2.5)
**Secondary outcomes**		
Walking behaviours – n (median, IQR)	64 (2, 1.75)	48 (4, 1.75)
Vigorous behaviours – n (median, IQR)	1 (0, 0)	3 (0, 0.75)
**Exploratory outcomes**		
Connect behaviours – n (median, IQR)	24 (0.5, 2)	17 (1.5, 2)
Take Notice behaviours – n (median, IQR)	5 (0, 0)	3 (0, 0.75)
Sedentary behaviours – n (median, IQR)	4 (0, 0)	2 (0, 0)

All values to two decimal places; *IQR* interquartile range

### Total number of people using the canal

Table 3 shows the results of the mixed-effects negative binomial regression models. Compared to the comparison group, the total number of people observed using the intervention canal path increased at all three follow-ups, controlling for day, time of day and precipitation: 7 months (IRR 1.67, 95% CI 1.44–1.95), 12 months (primary outcome) (IRR 2.10, 95% CI 1.79–2.48), and 24 months post-baseline (IRR 2.42, 95% CI 1.80–3.24). An IRR greater than 1 indicates an increase in the total number of people in the intervention group relative to the comparison group, whereas an IRR less than 1 indicates a decrease. For example, for the primary outcome, the IRR (2.10) at 12 months post-baseline suggests that the intervention is associated with a 110% increase in the total number of people compared to the comparison group.

**Table 3:** Mixed-effects negative binomial regression results for the total number of people. *Models adjusted for day, time of day and precipitation*

Time point	Total number of people (median per observation period)		Model results at each follow-up					
	Intervention group	Comparison group	Total number of observation periods in model	IRR	95% CI	Robust standard error	***p***-value	Random effect variance (site)
Baseline	51 (4)	65 (2)	–	–	–	–	–	–
7 months	148 (13.5)	117 (5)	72	1.67	1.44–1.95	0.13	< 0.001*	5.16e-34
12 months ^a^	142 (11.5)	88 (3)	72	2.10	1.79–2.48	0.17	< 0.001*	8.75e-34
24 months	154 (13.5)	99 (4)	70 ^b^	2.42	1.80–3.24	0.36	< 0.001*	5.76e-33

*CI* confidence interval, *IRR* incidence rate ratio

^a^ Primary outcome; ^b^ Two observation periods were cancelled at the intervention site due to ethical concerns regarding observer safety

*Statistically significant at *p* <  0.05 (z-test, two-tailed)

### Physical activity and other wellbeing behaviours

Table 4 shows the results for all physical activity levels and other wellbeing behaviours. Compared to the comparison group, Walking behaviour increased in the intervention site at all three follow-ups; Vigorous activity increased at 7 months (*p* = .009) and 24 months (*p* = .002), but not at 12 months (*p* = .96) post-baseline (Table 4); Connect behaviour increased at 12 months (*p* = .03) and 24 months (*p* = .006), but not at 7 months (*p* = .42) post-baseline (Table 4); and Take Notice behaviour increased at 24 months (*p* = .001), but not at 7 months (*p* = .07) or 12 months (*p* = .58) post-baseline (Table 4).

**Table 4:** Mann-Whitney U test results for all physical activity levels and other wellbeing behaviours

Outcome		Intervention group				Comparison group				Effect (difference between the change in the two groups)	***p***-value ^a^
		Number of observation periods, ***n***	Total count	Median (IQR)	Change in median (from baseline)	Number of observation periods, ***n***	Total count	Median (IQR)	Change in median (from baseline)		
Walking (secondary)	Baseline	12	48	4 (1.75)	–	24	64	2 (1.75)	–	–	–
	7 months	12	135	12.5 (8.5)	8.5	24	116	5 (3)	3	5.5	.005*
	12 months	12	140	11.5 (10)	7.5	24	88	3 (3)	1	6.5	< 0.001*
	24 months	10 ^b^	141	12 (9.5)	8	24	98	4 (3.75)	2	6	< 0.001*
Vigorous (secondary)	Baseline	12	3	0 (0.75)	–	24	1	0 (0)	–	–	–
	7 months	12	13	0.5 (2)	0.5	24	1	0 (0)	0	0.5	.009*
	12 months	12	7	0 (0.75)	0	24	1	0 (0)	0	0	.96
	24 months	10 ^b^	13	1 (2.25)	1	24	0	0 (0)	0	1	.002*
Sedentary (exploratory)	Baseline	12	2	0 (0)	–	24	4	0 (0)	–	–	–
	7 months	12	24	1.5 (4)	1.5	24	17	0 (1)	0	1.5	.07
	12 months	12	31	2 (1)	2	24	9	0 (0.75)	0	2	< 0.001*
	24 months	10 ^b^	24	2.5 (2.25)	2.5	24	8	0 (0)	0	2.5	< 0.001*
Connect (exploratory)	Baseline	12	17	1.5 (2)	–	24	24	0.5 (2)	–	–	–
	7 months	12	49	4 (5.25)	2.5	24	41	2 (2)	1.5	1	.42
	12 months	12	40	3 (5.75)	1.5	24	21	0 (2)	−0.5	2	.03*
	24 months	10 ^b^	49	3.5 (5.25)	2	24	25	0 (2)	−0.5	2.5	.006*
Take Notice (exploratory)	Baseline	12	3	0 (0.75)	–	24	5	0 (0)	–	–	–
	7 months	12	11	1 (1.75)	1	24	6	0 (0.75)	0	1	.07
	12 months	12	8	0 (1)	0	24	7	0 (0)	0	0	.58
	24 months	10 ^b^	15	1.5 (1)	1.5	24	3	0 (0)	0	1.5	.001*

*IQR* interquartile range

^a^ Mann-Whitney U tests were carried out to determine if there were significant differences in the change in counts of behaviours from baseline to each follow-up between intervention and comparison groups; ^b^ Two observation periods were cancelled at the intervention site due to ethical concerns regarding observer safety

* Statistically significant at *p* < 0.05 (z-test, two-tailed)

### Sensitivity analyses

Removing observation periods with high precipitation did not change the direction or statistical significance of the results for the secondary and exploratory outcomes (see Additional file 3).

### Process evaluation

#### Systematic observation

To assess whether displacement could account for the main findings, we assessed the impact of the intervention on use of the unchanged canal path. As shown in Table 5, the total number of people accessing the unchanged canal path decreased at 12 and 24 months post-baseline. This indicates there was some displacement to the intervention path from existing routes along the unchanged side of the canal. However, there was an overall increase in the combined total number of people using the canal at the intervention site (Table 5), which suggests that displacement from this particular route cannot explain all of the observed increases in use of the intervention path.

**Table 5:** Total number of people using the intervention path and unchanged path at the intervention site

Time point	Total number of people (median per observation period)		
	Intervention path	Unchanged path	Combined total
Baseline	51 (4)	103 (7)	154 (10.5)
7 months	148 (13.5)	143 (7.5)	291 (22.5)
12 months	142 (11.5)	88 (7)	230 (18.5)
24 months	154 (13.5)	63 (5)	217 (18)

#### Intercept surveys

A total of 53 participants completed intercept surveys, 58.9% of those approached: 21 in June/ July 2018 (7–8 months post-baseline); 15 in Nov/ Dec 2018 (12–13 months post-baseline); 17 in Nov/ Dec 2019 (24–25 months post-baseline). There were 30 females (56.6%) and 52 white participants (98.1%). The observed primary activity of these participants was walking (47.2%), dog walking (41.5%), running (7.5%), and cycling (3.8%). Most participants reported that they walked for at least 10 min at a time on several days a week (41.5%) or every day (39.6%) in the past month.

Twenty participants (37.7%) were already using the intervention path before the intervention was implemented. Thirty participants (56.6%) only started using the intervention path post-intervention: most participants (*n* = 23) were displaced from other existing routes along the canal, but some participants (*n* = 6) were new canal users; five of these six participants had moved house after the intervention.

Some participants reported that the intervention encouraged them to spend more time using the canal per visit (*n* = 10, 18.9%) or visit the canal more often per week (*n* = 3, 5.7%). Results of the intercept surveys are provided in Additional file 2.

## Discussion

### Key findings

The new walking infrastructure and green space improvements were associated with a statistically significant increase in the number of people using the intervention canal path compared to the comparison group at 7 months (IRR 1.67), 12 months (IRR 2.10) and 24 months (IRR 2.42) post-baseline. These increases were observed immediately at just one-month post-intervention (7 months post-baseline), with the largest effects observed at 18 months post-intervention (24 months post-baseline), suggesting that use of the canal path continued to increase over time. Examination of the IRR values suggests that these increases were sizable, especially at 12 and 24 months, where there were over twice as many people from baseline to follow-up using the intervention canal path relative to the comparison group i.e. IRR values greater than 2. The process evaluation suggested that there was displacement of activity from the existing unchanged canal path to the intervention path, but displacement from this particular canal route cannot explain all of the observed increases in use of the intervention path. Some survey participants reported that the intervention encouraged them to spend more time using the canal during each visit or visit the canal more often across the week.

There were some observed increases in walking and vigorous physical activity, social interactions, and people taking notice of the environment at the intervention canal path compared to the comparison group. The largest effects were observed on walking behaviour at all follow-ups, which is unsurprising given that the majority of people using the canal were walking rather than engaging in vigorous physical activity such as running or cycling. There were also notable increases in social interactions at 12 months and 24 months at the intervention path, with over twice as many social interactions compared to baseline (albeit from low baseline counts), whereas there were no observed changes in social interactions in the comparison group.

### Interpretation

There are two key reasons why improving access to this particular part of the canal may have caused increased use of this route. First, the resurfaced footpath and removal of encroaching vegetation provided better and safer access to ‘wild’ and interesting green corridors along the canal, thus providing a more pleasant walking and visual experience compared to the existing canal path. This accords with previous research which suggests that physical activity in natural environments is generally more enjoyable [56], and can encourage people to be active for longer and at higher intensities [57]. It is therefore plausible that the intervention may act as a catalyst to encourage and sustain physical activity.

Second, improving access to this side of the canal created a more attractive separate route for canal users (especially for people walking), which may have enabled better segregation of canal users engaging in different types of physical activity (e.g. walking, jogging, cycling), thus reducing shared space conflict. For example, some of the intercept survey participants who were walking reported that they started using the improved intervention path to avoid collisions with cyclists on the existing well-surfaced towpath on the other side of the canal. Previous research suggests that segregating pedestrians and cyclists on shared use routes is an effective way to reduce collisions and conflict [58], which is very relevant to canals paths as they are generally quite narrow but are used by a variety of users. It is also possible that reduction in shared space conflict between canal users may have contributed to the observed increases in social interactions at the intervention path 12 months and 24 months post-baseline, thus further enhancing the physical activity experience due to the increased enjoyment and wellbeing benefits associated with more social contact [59, 60]. In light of the current COVID-19 pandemic, the pertinence of reducing shared space conflict is heightened because of social distancing rules to reduce virus transmission [61].

Whilst this is the first natural experimental study of an urban canal intervention on physical activity to date, these findings are consistent with a similar recent controlled natural experimental study in the UK [62]. They found that a comparable intervention to improve access to urban woodlands (e.g. installing new footpaths, clearing overgrown vegetation) caused people to visit the woods more often, and increased physical activity levels, social cohesion and connectedness with nature. They also found that the intervention enhanced people’s experience of the woodlands, as shown by improvements in perceived restorativeness measures (e.g. spending time away from a day-to-day routine, fascination). These findings, together with the present study, suggest that interventions which improve access to existing urban green spaces, especially spaces that enable close physical and visual contact with natural features (e.g. trees, shrubs, water), are effective in causing positive behaviour change and improving people’s experience of urban green spaces. Importantly, the intervention providers in the present study included funding for 20 years of ongoing maintenance, which will increase the likelihood of providing long-term benefits and reduce negative effects on wellbeing associated with unmanaged green spaces (e.g. fear of crime) [63].

### Strengths and limitations

The practical and methodological challenges associated with evaluating natural experiments are well-documented [64–66]. This natural experimental study offers a feasible exemplar of how to address major methodological weaknesses causing high risk of bias in previous studies in this area. Key strengths of the present study include identifying multiple matched comparison sites using important objective variables (e.g. population density); using methods recommended by MRC guidance [18] to minimise the risk of confounding; conducting a sample size calculation; publication of a study protocol with a priori analyses specified and reporting any key deviations; clear reporting of samples and interventions in line with standardised checklists; and a mixed-methods approach. These are substantial improvements on many previous natural experimental studies that have often used poorly matched single comparison sites (rarely matched on objective variables); not controlled for key confounding factors by study design or in the analyses; not attempted sample size calculations; not published study protocols; often relied on single outcome measures (sometimes unvalidated); and are poorly reported [16, 17, 19].

We discovered the opportunity to evaluate the natural experiment just 6 weeks prior to implementation of the intervention. This demonstrates the feasibility of applying these robust methods even with short time frames to collect baseline data, which is crucial in the absence of suitable routinely collected data. Similar methods have been applied in two other natural experimental studies [48, 49].

Identifying closely matched comparison sites for environmental interventions is difficult, especially as these interventions tend to be implemented in a unique local context. Although we had to relax the matching criteria slightly (see Additional file 1), our newly developed process of comparison site matching provided well-matched sites (Table 1) and the samples were similar at baseline (Table 2). This matching process has been used in two other recent natural experimental studies [48, 49], therefore suggesting that this is a feasible method for future studies using matched intervention and comparison groups.

A process evaluation enabled us to strengthen causal inferences from the observations, including possible displacement effects; a key limitation of previous natural experimental studies in this area is not assessing for possible displacement effects [16]. However, we were unable to precisely estimate the effect of the intervention on net population-levels of physical activity, as we did not include measures that can assess within-person change over time (e.g. surveys, accelerometers). Nonetheless, within-person measures do suffer from certain methodological challenges (e.g. low response rates [19]), which is why triangulation of different outcome measures is optimal to strengthen causal inferences.

Statistical power was an issue for some of the wellbeing behaviours due to low counts (especially Vigorous and Take Notice behaviours), which prevented us from fitting appropriate regression models that could adjust for the total number of people and account for clustering. It is important to acknowledge that the Mann-Whitney U tests for each of the wellbeing behaviours could not account for clustering and therefore erroneously assumed that the outcomes were independent, which may have increased the risk of Type I errors (‘false positives’) due to artificially narrow confidence intervals [67]. Nevertheless, we powered the study for the primary outcome (i.e. total number of people), for which there was sufficient data to use regression modelling. MOHAWk data from this study will be valuable in informing sample size calculations and analytical plans in future studies of environmental interventions.

### Implications for policy and practice

We have provided robust practice-based evidence that urban canals offer promising settings for interventions to encourage green space usage and potentially enhance population health and wellbeing. This evidence will be particularly useful given increasing interest in evidence-informed urban design policies and practices [68, 69]. This study suggests that even relatively small-scale interventions which improve access to green corridors along canals and provide separate routes for different types of physical activities can have measurable impacts on the use of urban green spaces. However, current national UK policy and industry guidelines for planning and designing the physical environment to improve health and wellbeing do not emphasise the potential role of urban canals (e.g. [70–72]). This is an important gap as over 8 million people in England and Wales (14% of the population) live within 1 km of a canal or river [26]. There are also larger proportions of the population living near a canal or river in areas with high levels of deprivation in the UK, such as Greater Manchester (25% of the population) and West Midlands (51% of the population) [26]. Hence, urban canals have the potential to produce wide-reaching effects and address health inequalities.

Urban canals are particularly promising settings for urban green space interventions for a number of reasons. Canal paths offer pleasant recreational settings and also traffic-free utilitarian routes that connect suburban areas with city or town centres, as well as links between urban centres. They can facilitate a range of physical activities, including walking, jogging, cycling, and less physically exertive but increasingly popular ‘e-bikes’ and ‘e-scooters’ [73]. Canal paths tend to be predominantly flat, making them well suited to a multitude of users with varying physical abilities. Canals provide free access to green and blue space in dense built-up urban areas where proximity to local green and blue spaces may be limited, especially for populations that cannot access larger green and blue spaces such as national parks, coastlines and beaches. Crucially, as demonstrated in the present study, enhancement of a canal can increase use and only requires relatively small-scale change and maintenance, that can be readily replicated at low cost.

### Implications for research

The potential for urban canals to influence population health and wellbeing is understudied and the present research has suggested directions for future intervention research. Future studies should explore how urban canal interventions could reduce shared space conflict between different types of canal users. As well as finding ways to improve physical activity experiences of existing canal users, it is also important to examine the most effective interventions to encourage more new canal users from local communities, especially people from Black and Minority Ethnic (BAME) groups who are underrepresented users of canals [26], despite canals being more equally geographically distributed than parks and other urban green spaces [74]. A previous systematic review found that combining a physical change with a specific awareness/ promotion program (e.g. community events, outreach activities) is the most effective way to increase urban green space use and physical activity of users [16]. However, research needs to explore the most effective interventions that address specific barriers preventing use of urban canals (e.g. perceptions of safety [26]) and potential unintended consequences of such activities (e.g. exacerbating inequalities among BAME groups).

While the intervention in the present study seemed to be effective, more well-developed theory is needed to improve our understanding of whether findings can be generalised to different settings. In particular, it is important to understand how physical and social contexts impact intervention effectiveness, such as topography, deprivation and population demographics. A recent systematic review has begun to develop context-sensitive theory of how environmental interventions work to influence physical activity using realist evaluation methods [75]. This review found that environmental interventions that improve access to places or facilities (e.g. by installing new or improved walking routes) may provide an effective approach for increasing physical activity that is less sensitive to contextual factors. The results of this review therefore suggest that the intervention in the present study that improved access to the canal (e.g. by improving walking infrastructure) may also be effective in other contexts; for example, the intervention was implemented in a relatively affluent area, but the intervention may also be effective in more deprived areas. However, theoretical models need to incorporate other wellbeing behaviours beyond physical activity, especially social interactions given the role that these behaviours can have in supporting physical activity [76]. This mixed methods study provides robust evidence of intervention effectiveness required to advance theoretical understanding.

## Conclusions

Using rigorous natural experimental methods, this mixed-methods study shows that urban canals are promising settings for replicable small-scale interventions to increase green space usage and potentially increase physical activity and other wellbeing behaviours. This study has suggested avenues for future intervention research to further examine which urban canal interventions can effectively influence population health and wellbeing; including interventions that reduce shared space conflict, improve physical activity experiences for existing canal users and attract new canal users (particularly underrepresented populations). This study provides an exemplar to improve the internal validity of future natural experimental studies of environmental interventions, by addressing major methodological weaknesses causing high risk of bias in many previous natural experimental studies of environmental interventions.

## Supplementary Information

**Additional file 1.** Comparison site matching process.

**Additional file 2.** Intercept survey results.

**Additional file 3.** Sensitivity analyses.

**Additional file 4.** Completed TIDieR Checklist.

**Additional file 5.** Completed STROBE Checklist.

## Data Availability

Data are only available on reasonable request from the corresponding author. All materials required to use MOHAWk are freely available in Benton et al. [25]. Any other study materials are available by directly contacting the corresponding author on reasonable request.

## Abbreviations

ICC: Intraclass correlation coefficient
IMD: Index of Multiple Deprivation Score
IQR: Interquartile Range
IRR: Incidence Rate Ratio
JB: Jack Benton
LSOA: Lower Layer Super Output Area
MOHAWk: Method for Observing pHysical Activity and Wellbeing
MRC: Medical Research Council
NDVI: Normalised Difference Vegetation Index
NEF: New Economics Foundation
RCT: Randomised Controlled Trial
UK: United Kingdom
US: United States

## References

[1] World Health Organization. Urban Green Space Interventions and Health: A review of impacts and effectiveness. Copenhagen; 2017. Available from: http://www.euro.who.int/__data/assets/pdf_file/0010/337690/FULL-REPORT-for-LLP.pdf?ua=1

[2] Lee ACK, Maheswaran R (2010). The health benefits of urban green spaces: a review of the evidence. J Public Health.

[3] Houlden V, Weich S, Porto de Albuquerque J, Jarvis S, Rees K (2018). The relationship between greenspace and the mental wellbeing of adults: A systematic review. PLoS One.

[4] Bell SL, Phoenix C, Lovell R, Wheeler BW (2014). Green space, health and wellbeing: making space for individual agency. Health Place.

[5] United Nations (2015). World urbanization prospects: the 2014 revision.

[6] World Health Organization (2016). Urban green spaces and health: a review of evidence.

[7] Markevych I, Schoierer J, Hartig T, Chudnovsky A, Hystad P, Dzhambov AM (2017). Exploring pathways linking greenspace to health: theoretical and methodological guidance. Environ Res.

[8] Warburton DER, Nicol CW, Bredin SSD (2006). Health benefits of physical activity: the evidence. CMAJ.

[9] Penedo FJ, Dahn JR (2005). Exercise and well-being: a review of mental and physical health benefits associated with physical activity. Curr Opin Psychiatry.

[10] Pretty JN, Barton J (2005). The mental and physical health outcomes of green exercise. Int J Environ Health Res.

[11] Maas J, van Dillen SME, Verheij RA, Groenewegen PP (2009). Social contacts as a possible mechanism behind the relation between green space and health. Health Place.

[12] Helliwell JF, Putnam RD, Huppert FA, Baylis N, Keverne B (2005). The social context of well-being. The science of well-being.

[13] Anderson J, Ruggeri K, Steemers K, Huppert F (2017). Lively social space, well-being activity, and Urban Design: findings from a low-cost community-led public space intervention. Environ Behav.

[14] McEwan K, Richardson M, Sheffield D, Ferguson FJ, Brindley P (2019). A smartphone app for improving mental health through connecting with urban nature. Int J Environ Res Public Health.

[15] Seligman MEP, Steen TA, Peterson C (2005). Positive psychology Progress: empirical validation of interventions. Am Psychol.

[16] Hunter RF, Christian H, Veitch J, Astell-Burt T, Hipp JA, Schipperijn J (2015). The impact of interventions to promote physical activity in urban green space: a systematic review and recommendations for future research. Soc Sci Med.

[17] World Health Organization. Urban Green Space Interventions and Health: A review of evidence. Copenhagen; 2017. Available from: http://www.euro.who.int/__data/assets/pdf_file/0005/321971/Urban-green-spaces-and-health-review-evidence.pdf?ua=1

[18] Craig P, Gunnell D, Ogilvie D, Sutton M, Thompson S (2012). Using natural experiments to evaluate population health interventions: guidance for producers and users of evidence prepared on behalf of the Medical Research Council.

[19] Benton JS, Anderson J, Hunter RF, French DP. The effect of changing the built environment on physical activity: a quantitative review of the risk of bias in natural experiments. Int J Behav Nutr Phys Act. 2016;13:107. 10.1186/s12966-016-0433-3 PMC5055702 27717360

[20] Roberts H, McEachan R, Margary T, Conner M, Kellar I (2018). Identifying effective behavior change techniques in built environment interventions to increase use of green space: a systematic review. Environ Behav.

[21] Kärmeniemi M, Lankila T, Ikäheimo T, Koivumaa-Honkanen H, Korpelainen R (2018). The built environment as a determinant of physical activity: a systematic review of longitudinal studies and natural experiments. Ann Behav Med.

[22] MacMillan F, George ES, Feng X, Merom D, Bennie A, Cook A, et al. Do natural experiments of changes in neighborhood built environment impact physical activity and diet? A systematic review. Int J Environ Res Public Health. 2018;15(2):217–29. 10.3390/ijerph15020217 PMC5858286 29373567

[23] Sallis JF, Cerin E, Conway TL, Adams MA, Frank LD, Pratt M (2016). Physical activity in relation to urban environments in 14 cities worldwide: a cross-sectional study. Lancet..

[24] New Economics Foundation (2008). Five ways to wellbeing.

[25] Benton JS, Anderson J, Pulis M, Cotterill S, Hunter RF, French DP. Method for observing pHysical activity and wellbeing (MOHAWk): validation of an observation tool to assess physical activity and other wellbeing behaviours in urban spaces. Cities Health. 2020:1–15.

[26] Canal & River Trust (2017). Waterways & Wellbeing. Building the Evidence Base.

[27] Gascon M, Zijlema W, Vert C, White MP, Nieuwenhuijsen MJ (2017). Outdoor blue spaces, human health and well-being: a systematic review of quantitative studies. Int J Hyg Environ Health.

[28] Canal & River Trust. Annual Report & Accounts 2018/19. Milton Keynes; 2019. Available from: https://canalrivertrust.org.uk/media/original/40488-annual-report-and-accounts-2018-2019.pdf?v=f4ac07

[29] Scottish Canals (2020). Funding boost keeps Scotland’s canals flowing into the future.

[30] European Environment Agency. Rivers and lakes in European cities—past and future challenges. Copenhagen; 2016. Available from: https://www.eea.europa.eu/publications/rivers-and-lakes-in-cities.

[31] Benton J, Anderson J, Cotterill S, Dennis M, French DP. The impact of new walking infrastructure and changes to green space along an urban canal on physical activity and wellbeing: protocol for a natural experimental study. Open Sci Frame. 2018; Available from: https://osf.io/zcm7v/. 10.1186/s12889-018-5812-z PMC6062989 30053861

[32] Salford City Council (2015). Indices of Deprivation 2015 - the City of Salford’s position.

[33] Salford City Council (2020). Boothstown and Ellenbrook Ward profile - January 2020..

[34] Peel Land and Property Group (2017). Improvements to green spaces and recreational routes in Boothstown Salford.

[35] Cain KL, Gavand KA, Conway TL, Geremia CM, Millstein RA, Frank LD (2017). Developing and validating an abbreviated version of the microscale audit for pedestrian streetscapes (MAPS-abbreviated). J Transp Health.

[36] Gidlow CJ, Ellis NJ, Bostock S (2012). Development of the Neighbourhood green space tool (NGST). Landsc Urban Plan.

[37] Benton JS, Anderson J, Cotterill S, Dennis M, Lindley SJ, French DP. Evaluating the impact of improvements in urban green space on older adults’ physical activity and wellbeing: protocol for a natural experimental study. BMC Public Health. 2018;18(923):1–15. 10.1186/s12889-018-5812-z PMC6062989 30053861

[38] Humpel N, Owen N, Leslie E (2002). Environmental factors associated with adults’ participation in physical activity. A review. Am J Prevent Med.

[39] Owen N, Humpel N, Leslie E, Bauman A, Sallis JF (2004). Understanding environmental influences on walking: Review and research agenda. Am J Prevent Med.

[40] Duncan MJ, Spence JC, Mummery WK (2005). Perceived environment and physical activity: A meta-analysis of selected environmental characteristics. Int J Behav Nutr Phys Act.

[41] Wendel-Vos W, Droomers M, Kremers S, Brug J, Van Lenthe F (2007). Potential environmental determinants of physical activity in adults: a systematic review. Obes Rev.

[42] Saelens BE, Handy SL (2008). Built environment correlates of walking: a review. Med Sci Sports Exerc.

[43] Panter JR, Jones A (2010). Attitudes and the environment as determinants of active travel in adults: what do and don’t we know?. J Phys Act Health.

[44] Moran M, Van Cauwenberg J, Hercky-Linnewiel R, Cerin E, Deforche B, Plaut P (2014). Understanding the relationships between the physical environment and physical activity in older adults: a systematic review of qualitative studies. Int J Behav Nutr Phys Act.

[45] Barnett DW, Barnett A, Nathan A, Van Cauwenberg J, Cerin E. Built environmental correlates of older adults’ total physical activity and walking: a systematic review and meta-analysis. Int J Behav Nutr Phys Act. 2017;14(1):103. 10.1186/s12966-017-0558-z PMC5547528 28784183

[46] Cerin E, Nathan A, van Cauwenberg J, Barnett DW, Barnett A (2017). The neighbourhood physical environment and active travel in older adults: a systematic review and meta-analysis. Int J Behav Nutr Phys Act.

[47] Department for Communities and Local Government (2015). English Indices of Deprivation.

[48] Benton JS, Cotterill S, Anderson J, Macintyre VG, Gittins M, Dennis M, et al. Impact of a low-cost urban green space intervention on wellbeing behaviours in older adults: a natural experimental study. Wellbeing, Space & Society. In press.

[49] Anderson J, French DP, Benton JS, Dennis M, Rothwell J. The effects of the grow green urban greening improvements on physical health and wellbeing behaviours in Manchester UK: a natural experimental study. Open Sci Frame. 2020; Available from: https://osf.io/zqgcn.

[50] Cohen DA, Setodji C, Evenson KR, Ward P, Lapham S, Hillier A (2011). How much observation is enough? Refining the administration of SOPARC. J Phys Act Health.

[51] Koo TK, Li MY (2016). A guideline of selecting and reporting Intraclass correlation coefficients for reliability research. J Chiropr Med.

[52] Panter J, Guell C, Prins R, Ogilvie D (2017). Physical activity and the environment: conceptual review and framework for intervention research. Int J Behav Nutr Phys Act.

[53] Donner A, Klar N. Design and analysis of cluster randomization trials in health research. London: Wiley; 2000. p. 178.

[54] Cameron AC, Trivedi PK. Regression analysis of count data. Cambridge: Cambridge University Press; 1998.

[55] Craig P, Vittal Katikireddi S, Leyland A, Popham F (2017). Natural experiments: an overview of methods, approaches, and contributions to public health intervention research. Annu Rev Public Health.

[56] Gladwell VF, Brown DK, Wood C, Sandercock GR, Barton JL (2013). The great outdoors: How a green exercise environment can benefit all. Extrem Physiol Med.

[57] Lahart I, Darcy P, Gidlow C, Calogiuri G (2019). The effects of green exercise on physical and mental wellbeing: A systematic review. Int J Environ Res Public Health.

[58] Atkins (2012). Shared Use: Operational Review.

[59] Howick J, Kelly P, Kelly M (2019). Establishing a causal link between social relationships and health using the Bradford Hill guidelines. SSM Popul Health.

[60] Francis J, Giles-Corti B, Wood LJ, Wood L, Knuiman M. Creating sense of community: the role of public space. J Environ Psychol. 2012;32:401–9.

[61] De Vos J (2020). The effect of COVID-19 and subsequent social distancing on travel behavior. Transp Res Interdiscip Perspect.

[62] Ward Thompson C, Silveirinha de Oliveira E, Tilley S, Elizalde A, Botha W, Briggs A (2019). Health impacts of environmental and social interventions designed to increase deprived communities’ access to urban woodlands: a mixed-methods study. Public Health Res.

[63] Jorgensen A, Hitchmough J, Calvert T (2002). Woodland spaces and edges: their impact on perception of safety and preference. Landsc Urban Plan.

[64] Crane M, Bohn-Goldbaum E, Grunseit A, Bauman A (2020). Using natural experiments to improve public health evidence: a review of context and utility for obesity prevention. Health Res Policy Syst.

[65] Ogilvie D, Adams J, Bauman A, Gregg EW, Panter J, Siegel KR (2019). Using natural experimental studies to guide public health action: turning the evidence-based medicine paradigm on its head. J Epidemiol Commun Health.

[66] Veitch J, Salmon J, Giles-Corti B, Crawford D, Dullaghan K, Carver A (2017). Challenges in conducting natural experiments in parks—lessons from the REVAMP study. Int J Behav Nutr Phys Act.

[67] Clarke P (2008). When can group level clustering be ignored? Multilevel models versus single-level models with sparse data. J Epidemiol Community Health.

[68] Le Gouais A, Foley L, Ogilvie D, Guell C. Decision-making for active living infrastructure in new communities: a qualitative study in England. J Public Health. 2019;105:e249–e258. 10.1093/pubmed/fdz105 PMC7435215 31565741

[69] Public Health England. Improving access to greenspace: A new review for 2020. London; 2020. Available from: https://assets.publishing.service.gov.uk/government/uploads/system/uploads/attachment_data/file/904439/Improving_access_to_greenspace_2020_review.pdf.

[70] Public Health England (2017). Spatial Planning for Health An evidence resource for planning and designing healthier places.

[71] National Institute for Health and Clinical Excellence (NICE) (2018). Physical activity and the environment.

[72] Ministry of Housing C& LG. National Design Guide. In: Planning practice guidance for beautiful, enduring and successful places. London; 2019. Available from: https://www.gov.uk/government/publications/national-design-guide.

[73] Fishman E, Cherry C. E-bikes in the mainstream: reviewing a decade of research E-bikes in the mainstream: reviewing a decade of research. Transp Rev. 2015;36(1):72–91.

[74] Tieges Z, McGregor D, Georgiou M, Smith N, Saunders J, Millar R (2020). The impact of regeneration and climate adaptations of urban green–blue assets on all-cause mortality: a 17-year longitudinal study. Int J Environ Res Public Health.

[75] Panter J, Guell C, Humphreys D, Ogilvie D (2019). Can changing the physical environment promote walking and cycling? A systematic review of what works and how. Health Place.

[76] McNeill LH, Kreuter MW, Subramanian SV (2006). Social environment and physical activity: a review of concepts and evidence. Soc Sci Med.

