# Supplementary material for: A natural experimental study of improvements along an urban canal: impact on canal usage, physical activity and other wellbeing behaviours
Source: Int J Behav Nutr Phys Act. 2021 Jan 27;18:19. doi: 10.1186/s12966-021-01088-w (PMC7838466; doi:10.1186/s12966-021-01088-w)
Supplement: Supplementary file 1 — Additional file 1. Comparison site matching process. [file 12966_2021_1088_MOESM1_ESM.docx]

**Additional file 2. Comparison site matching process**

The overall aim of the comparison site matching process was to identify two closely matched comparison sites for the intervention site, using several key objective and subjective environmental correlates of physical activity.

***Step one: Matching at the neighbourhood (LSOA) level***

The intervention site is located along a canal waterway, so the overall aim of the process was to identify the most closely matched waterways based on key variables; comparison sites could then be identified within these waterways. The first step was to identify the most closely matched neighbourhoods in which the potential waterways for comparison sites could be searched from.

Due to a lack of available walkability indices for Greater Manchester (GM) it was necessary to manually search for neighbourhoods based on available spatial data at the Lower Layer Super Output Area (LSOA) level (census reporting units containing between 1000 and 3000 individuals) [1,2]. Population density, defined as the number of persons per hectare, was used as a proxy measure of residential density. Street connectivity was measured using street intersection density; the number of 3-way junctions standardised by LSOA area. Normalised Difference Vegetation Index (NDVI) scores, a normalised scale of healthy vegetation cover [3], were used for presence of greenery. Socioeconomic status was measured using the Index of Multiple Deprivation (IMD) Score [4]; an area deprivation score that combines several indicators of deprivation including income, employment, health and crime. Spatial analyses were carried out using ArcGIS 10.4.

To identify the most closely matched LSOAs to the intervention LSOA, a systematic funneling approach was used. All LSOAs in GM were firstly ranked in order of residential density and the 100 most closely matched LSOAs to each intervention site LSOA were extracted. These 100 LSOAs were then ranked in order of street connectivity and the 50 most closely matched LSOAs were selected. These two variables were matched first because they are the strongest and most consistent correlates of physical activity [5]. Next, the remaining 50 LSOAs were ranked in order of closeness of IMD score to the intervention LSOA and the most closely matched 25 LSOAs were selected. Finally, these 25 LSOAs were ranked in order of those most closely matched on NDVI scores to the intervention LSOA and the five most closely matched LSOAs were selected. Thus, by the end of this step there were a manageable total of five potential LSOAs for each intervention site.

***Step two: Matching at the site level (access to/ availability of destinations and services)***

As there were no data available for ‘access to/ availability of destinations and services’ at the LSOA level, the second step was to measure this variable at the site level. There is a lack of data on walkability scores in GM and manually calculating distances to nearby destinations and services for each site within each LSOA was beyond the scope of this project. Instead, Walk Score (www.walkscore.com) was the most appropriate objective and reliable measure readily available. Walk Score is a website that uses a Google search algorithm to calculate a weighted score (1-100) based on the number and accessibility of amenities (such as shops and parks) within a 1-mile radius of a user-entered postcode, whereby closer amenities with the most accessible walking routes are weighted more strongly. Walk Scores have shown good correlation with gold-standard measures of walkability using Geographic Information Systems (GIS) [6] and have previously been used in studies in the US (e.g. [7]). Walk Score confirmed that data is available in in the UK (WalkScore, personal communication).

To calculate Walk Scores for each site in each LSOA, it was necessary to obtain all postcodes within each LSOA. Postcodes were obtained using FreeMapTools (www.freemaptools.com): a free website that enables users to search for all postcodes within a user-defined area in the UK. Walk Scores were then calculated for each intervention site postcode and all postcodes extracted from the potential comparison site LSOAs. All postcodes were then ranked in order of closeness of Walk Score to each intervention site.

There were difficulties in identifying closely matched comparison sites. To increase the number of potential matches, we considered all types of inland linear waterways which may serve a similar recreational function as canals (e.g. rivers, brooks); which produced the first suitable comparison site (Comparison site 1A).

***Step three: Matching at the site level (site level characteristics)***

The aim of step three was to find at least three potential comparison sites located within sites (postcodes) most closely matched to the intervention site based on site level characteristics. All postcodes were remotely audited using Google Maps and, where possible, Google Street View. Google Street View can be accessed via Google Maps ([www.maps.google.com](http://www.maps.google.com)) and permits users to remotely navigate 360° through panoramic images of the environment from the internet. Virtual environmental audits were preferred given the vast number of postcodes that needed to be examined. Empirical research has previously demonstrated that Google Street View is mostly a reliable and efficient tool to measure the streetscape in comparison with physical on-site audits [8].

Starting with postcodes most closely matched to the intervention site based on Walk Scores, JB audited the following objective streetscape characteristics associated with adults’ and older adults’ physical activity for each postcode: type of road nearest to the waterway (proxy measure of traffic), presence of a footpath and footbridge, number of pedestrian access points to the waterway (proxy measure of connectivity), number of benches, presence of lighting, presence of greenery, and non-residential buildings. For some of the intervention sites, it was necessary to iteratively search for more potential comparison sites; this was done by identifying more LSOAs and repeating the first three steps. By the end of step three, the intervention site had only one potential comparison site

To identify more potential comparison sites, the same matching process was used but potential neighbourhoods for step one was purposefully identified from the same canal route as the intervention site, within the boundaries of Greater Manchester. This resulted in a second potential comparison site.

***Step four: On-site environmental audits***

The aim of step four was to ensure the two potential comparison sites were closely matched in terms of quality and quantity of green space and footpaths. These variables are more difficult to reliably judge using Google Street View and therefore required on-site audits of the environment.

JB visited each potential comparison site to systematically audit each site using two validated environmental audit tools: the Neighbourhood Green Space Tool (NGST) [9] and the 54-item abbreviated version of the Microscale Audit of Pedestrian Streetscapes (MAPS-Abbreviated) measure of street design [10]. NGST enabled an audit of the green space characteristics and was specifically developed in the UK; which was important due to the lack of environmental walkability tools that have been developed in the UK [11]. MAPS-Abbreviated was chosen because it measures the quality of pedestrian footpaths and was partly based on a previous tool that has been modified by the Healthy Aging Network [12]. The scores on both tools in each potential comparison site and intervention site were used to ensure the two potential comparison sites were closely matched to the intervention site.

***Step five: Matching on pedestrian traffic***

The fifth and final step aimed to ensure that intervention and comparison sites were closely matched in terms of pedestrian traffic; that is, the frequency of users passing through a site. It has been found that 15 minutes of observation can provide excellent reliability in estimating the frequency of users passing through a site across the whole hour [13]. Therefore, 15-minute observations were carried out across the three sites (one intervention and two comparison sites) during November 2017 to count pedestrian traffic. JB conducted 15-minute observations at the intervention site and one comparison site on a Friday between 10am-12pm. Due to time restraints, JB conducted 15 minute observations at the other comparison site on a different day (Monday) between 10am-12pm. All final comparison sites were similar to the intervention site in terms of pedestrian traffic and subjective ‘feel’ of the sites.

***Difficulties identifying multiple closely matched comparison sites***

We could only identify one closely matched comparison site using this rigorous matching process. To identify a second comparison site, the same matching process was used but potential neighbourhoods in step one was identified from the same canal as the intervention site (i.e. Bridgewater Canal) within Greater Manchester, therefore not matching on neighbourhood level variables. Comparison site 1B was approximately 8.8 km walking distance from the intervention site and there are no direct footpath links between the sites, thus reducing the risk of contamination between intervention and comparison sites.

**References**

1. Office for National Statistics. 2001 Census: Digitised Boundary Data (England and Wales) [computer file]. UK Data Service Census Support. Downloaded from: http://edina.ac.uk/census. Licensed under the terms of the Open Government Licence [http://www.nationalarchives.gov.uk/doc/open-government-licence/ version/2]
2. Office for National Statistics. Super Output Areas: Office for National Statistics, 2011
3. Markevych I, Schoierer J, Hartig T, Chudnovsky A, Hystad P, Dzhambov AM, De Vries S, Triguero-Mas M, Brauer M, Nieuwenhuijsen MJ, Lupp G. Exploring pathways linking greenspace to health: Theoretical and methodological guidance. Environ Res. 2017;158:301-17.
4. Department for Communities and Local Government. 2015. English Indices of Deprivation 2015 [computer file]. Downloaded from: https://www.gov.uk/government/statistics/english-indices-of-deprivation-2015. Licensed under: <https://www.nationalarchives.gov.uk/doc/open-government-licence/version/3>
5. Bauman AE, Reis RS, Sallis JF, Wells JC, Loos RJ, Martin BW. Correlates of physical activity: why are some people physically active and others not? Lancet. 2012;380(9838):258–71.
6. Carr LJ, Dunsiger SI, Marcus BH. Validation of Walk Score for estimating access to walkable amenities. Br J Sports Med. 2011;45(14):1144-8.
7. Althoff T, Sosič R, Hicks JL, King AC, Delp SL, Leskovec J. Large-scale physical activity data reveal worldwide activity inequality. Nature. 2017;547(7663):336-9.
8. Badland HM, Opit S, Witten K, Kearns RA, Mavoa S. Can virtual streetscape audits reliably replace physical streetscape audits?. J Urban Health. 2010;87(6):1007-16.
9. Gidlow CJ, Ellis NJ, Bostock S. Development of the neighbourhood green space tool (NGST). Landsc and Urban Planning. 2012;106(4):347-58.
10. Cain KL, Gavand KA, Conway TL, Geremia CM, Millstein RA, Frank LD, Saelens BE, Adams MA, Glanz K, King AC, Sallis JF. Developing and validating an abbreviated version of the Microscale Audit for Pedestrian Streetscapes (MAPS-Abbreviated). J Transport & Health. 2017; 5: 84-96.
11. Brownson RC, Hoehner CM, Day K, Forsyth A, Sallis JF. Measuring the built environment for physical activity: state of the science. Am J Prev Med. 2009;36(4):S99-123.
12. Kealey M, Kruger J, Hunter R, Ivey S, Satariano W, Bayles C, Ramirez B, Bryant L, Johnson C, Lee C, Levinger D. Engaging older adults to be more active where they live: audit tool development. Prev Chronic Dis. 2005; 2:1–2.
13. Benton JS, Anderson J, Pulis M, Cotterill S, Hunter RF, French DP. Method for Observing pHysical Activity and Wellbeing (MOHAWk): validation of an observation tool to assess physical activity and other wellbeing behaviours in urban spaces. Cities Health. 2020.
