# Supplementary material for: A natural experimental study of improvements along an urban canal: impact on canal usage, physical activity and other wellbeing behaviours
Source: Int J Behav Nutr Phys Act. 2021 Jan 27;18:19. doi: 10.1186/s12966-021-01088-w (PMC7838466; doi:10.1186/s12966-021-01088-w)
Supplement: Supplementary file 2 — Additional file 2. Intercept survey results. [file 12966_2021_1088_MOESM2_ESM.docx]

**Additional file 2.** Main results of the intercept surveys.

| **Question** | **Response options** | **Frequencies** ^a^  **(% of total eligible)** |
| --- | --- | --- |
| When was the first time you started using this particular path along the canal? | Nov 2017 or earlier  (pre-intervention)  May 2018 or later  (post-intervention) | 20 (37.7%)  33 (62.3%) |
| [Of those who started using canal path after the intervention] Why did you start using this particular path along the canal? | Because of recent changes  Moved house  Other | 27 (81.8%)  5 (15.2%)  1 (3%) |
| Change in canal usage | Always used intervention path (no change)  Started using new canal path after the intervention changes, but displaced from elsewhere on canal (displacement)  Started using new canal path after the intervention changes (new canal users)  Did not answer | 20 (37.7%)  23 (43.4%)  7 (13.2%)  3 (5.7%) |
| Have you noticed any recent physical changes to this particular canal path and the areas around the canal? | Yes  No | 47 (88.7%)  6 (11.3%) |
| Do you think these changes have encouraged you to visit the canal more often per week? | Strongly agree  Slightly agree  Neither agree nor disagree  Slightly disagree  Strongly disagree  Did not answer | 1 (1.9%)  2 (3.8%)  20 (37.7%)  13 (24.6%)  12 (22.6%)  5 (9.4%) |
| Do you think these changes have encouraged you to spend more time using the canal per visit? | Strongly agree  Slightly agree  Neither agree nor disagree  Slightly disagree  Strongly disagree  Did not answer | 3 (5.7%)  7 (13.2%)  13 (24.5%)  10 (18.9%)  15 (28.3%)  5 (9.4%) |
| ^a^ There was a total of 53 participants | | |
