# Supplementary material for: A natural experimental study of improvements along an urban canal: impact on canal usage, physical activity and other wellbeing behaviours
Source: Int J Behav Nutr Phys Act. 2021 Jan 27;18:19. doi: 10.1186/s12966-021-01088-w (PMC7838466; doi:10.1186/s12966-021-01088-w)
Supplement: Supplementary file 3 — Additional file 3. Sensitivity analyses. [file 12966_2021_1088_MOESM3_ESM.docx]

**Additional file 3.** Median counts of wellbeing behaviours, with high precipitation observation periods removed ^a^

| **Outcome** | | **Intervention observation periods (*n* = 46)** | | | **Comparison observation periods (*n* = 96)** | | | **Effect**  **(difference between the change in the two groups)** | ***p*-value** ^a^ |
| --- | --- | --- | --- | --- | --- | --- | --- | --- | --- |
|  |  | **Median (IQR)** | **Total** | **Change in median (from baseline)** | **Median (IQR)** | **Total** | **Change in median (from baseline)** |  |  |
| Walking  (secondary) | Baseline | 4 (1.75) | 48 | - | 2 (1) | 47 | - | - | - |
|  | 7 months | 12.5 (8.5) | 135 | 8.5 | 5 (3) | 116 | 3 | 5.5 | .004* |
|  | 12 months | 11.5 (10) | 140 | 7.5 | 5 (4.5) | 71 | 3 | 4.5 | .002* |
|  | 24 months | 12 (10.5) | 135 | 8 | 4 (4) | 64 | 2 | 6 | < 0.001* |
| Vigorous  (secondary) | Baseline | 0 (0.75) | 3 | - | 0 (0) | 1 | - | - | - |
|  | 7 months | 0.5 (2) | 13 | 0.5 | 0 (0) | 1 | 0 | 0.5 | .02* |
|  | 12 months | 0 (0.75) | 7 | 0 | 0 (0) | 1 | 0 | 0 | .95 |
|  | 24 months | 1 (2) | 13 | 1 | 0 (0) | 0 | 0 | 1 | .01* |
| Sedentary  (exploratory) | Baseline | 0 (0) | 2 | - | 0 (0) | 3 | - | - | - |
|  | 7 months | 1.5 (4) | 24 | 1.5 | 0 (1) | 17 | 0 | 1.5 | .07 |
|  | 12 months | 2 (1) | 31 | 2 | 0 (1) | 8 | 0 | 2 | < 0.001* |
|  | 24 months | 3 (1.5) | 24 | 3 | 0 (1) | 8 | 0 | 3 | < 0.001* |
| Connect  (exploratory) | Baseline | 1.5 (2) | 17 | - | 1 (2) | 22 | - | - | - |
|  | 7 months | 4 (5.25) | 49 | 2.5 | 2 (2) | 41 | 1 | 1.5 | .24 |
|  | 12 months | 3 (5.75) | 40 | 1.5 | 1 (2) | 19 | 0 | 1.5 | .04* |
|  | 24 months | 5 (6.5) | 49 | 3.5 | 0 (2) | 18 | -1 | 4.5 | .001* |
| Take Notice  (exploratory) | Baseline | 0 (0.75) | 3 | - | 0 (0) | 5 | - | - | - |
|  | 7 months | 1 (1.75) | 11 | 1 | 0 (0.75) | 6 | 0 | 1 | .052 |
|  | 12 months | 0 (1) | 8 | 0 | 0 (1) | 7 | 0 | 0 | .60 |
|  | 24 months | 2 (1) | 15 | 2 | 0 (0) | 3 | 0 | 2 | < 0.001* |
| ^a^ Mann-Whitney *U* tests were carried out to determine if there were significant differences in the change in counts of behaviours from baseline between intervention and comparison groups; * Statistically significant at p < 0.05 (z-test, two-tailed)  *IQR* interquartile range | | | | | | | | | |
