# Supplementary material for: A natural experimental study of improvements along an urban canal: impact on canal usage, physical activity and other wellbeing behaviours
Source: Int J Behav Nutr Phys Act. 2021 Jan 27;18:19. doi: 10.1186/s12966-021-01088-w (PMC7838466; doi:10.1186/s12966-021-01088-w)
Supplement: Supplementary file 4 — Additional file 4. Completed TIDieR Checklist. [file 12966_2021_1088_MOESM4_ESM.docx]

**
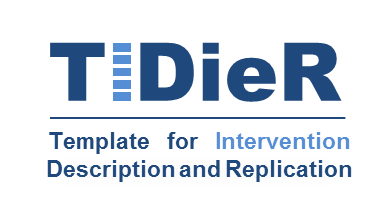
The TIDieR (Template for Intervention Description and Replication) Checklist*:**

Information to include when describing an intervention and the location of the information

| **Item number** | **Item** | **Where located **** | |
| --- | --- | --- | --- |
|  |  | Primary paper  (page or appendix  number) | Other ^†^ (details) |
|  | **BRIEF NAME** |  |  |
| **1.** | Provide the name or a phrase that describes the intervention. | Urban canal improvements (page 7) | ______________ |
|  | **WHY** |  |  |
| **2.** | Describe any rationale, theory, or goal of the elements essential to the intervention. | The intervention providers believed this would be an effective intervention to “enhance existing recreational provision which is used by the local community and the key connections between these recreational spaces. The improvements will enable and encourage circular walks to be undertaken by the local community in Boothstown. The improvements will help in creating pleasant routes for recreation and exercise to promote health and wellbeing, while also increasing biodiversity.” (page 9) | _____________ |
|  | **WHAT** |  |  |
| **3.** | Materials: Describe any physical or informational materials used in the intervention, including those provided to participants or used in intervention delivery or in training of intervention providers. Provide information on where the materials can be accessed (e.g. online appendix, URL). | The interventions included new footpaths; resurfacing of existing footpaths using golden gravel; enhancement of an existing nature park and village green (e.g. improved entrance points); clearance and management of vegetation; new benches, signage and informal play equipment (page 9) | _____________ |
| **4.** | Procedures: Describe each of the procedures, activities, and/or processes used in the intervention, including any enabling or support activities. | N/A | _____________ |
|  | **WHO PROVIDED** |  |  |
| **5.** | For each category of intervention provider (e.g. psychologist, nursing assistant), describe their expertise, background and any specific training given. | The intervention was designed, funded and implemented by Peel Land and Property Group (page 9) | _____________ |
|  | **HOW** |  |  |
| **6.** | Describe the modes of delivery (e.g. face-to-face or by some other mechanism, such as internet or telephone) of the intervention and whether it was provided individually or in a group. | N/A - the interventions were physical changes to the environment, so there was not necessarily a ‘mode of delivery’ per se. The interventions were designed and funded by Peel Land and Property Group. (page 9) | _____________ |
|  | **WHERE** |  |  |
| **7.** | Describe the type(s) of location(s) where the intervention occurred, including any necessary infrastructure or relevant features. | The intervention was implemented in the city of Salford, which is the 22nd most deprived local authority in England. However, the intervention was located in an affluent area within Salford called Boothstown and Ellensbrook (population = 9,532), which is the 2nd least deprived ward in Salford (page 8) | _____________ |
|  | **WHEN and HOW MUCH** |  |  |
| **8.** | Describe the number of times the intervention was delivered and over what period of time including the number of sessions, their schedule, and their duration, intensity or dose. | The interventions were implemented in December 2017 – May 2018, and a smaller second intervention phase in February 2019 (page 9) | _____________ |
|  | **TAILORING** |  |  |
| **9.** | If the intervention was planned to be personalised, titrated or adapted, then describe what, why, when, and how. | N/A – these were physical changes to the environment, and therefore changes were the same for everyone. | _____________ |
|  | **MODIFICATIONS** |  |  |
| **10.^ǂ^** | If the intervention was modified during the course of the study, describe the changes (what, why, when, and how). | The interventions were delivered as planned (page 9) | _____________ |
|  | **HOW WELL** |  |  |
| **11.** | Planned: If intervention adherence or fidelity was assessed, describe how and by whom, and if any strategies were used to maintain or improve fidelity, describe them. | The interventions were delivered as planned (page 9) | _____________ |
| **12.^ǂ^** | Actual: If intervention adherence or fidelity was assessed, describe the extent to which the intervention was delivered as planned. | We took photos of the intervention components at follow-up to demonstrate the interventions post-implementation (page 13) | _____________ |

** **Authors** - use N/A if an item is not applicable for the intervention being described. **Reviewers** – use ‘?’ if information about the element is not reported/not sufficiently reported.

† If the information is not provided in the primary paper, give details of where this information is available. This may include locations such as a published protocol or other published papers (provide citation details) or a website (provide the URL).

ǂ If completing the TIDieR checklist for a protocol, these items are not relevant to the protocol and cannot be described until the study is complete.

* We strongly recommend using this checklist in conjunction with the TIDieR guide (see *BMJ* 2014;348:g1687) which contains an explanation and elaboration for each item.

* The focus of TIDieR is on reporting details of the intervention elements (and where relevant, comparison elements) of a study. Other elements and methodological features of studies are covered by other reporting statements and checklists and have not been duplicated as part of the TIDieR checklist. When a **randomised trial** is being reported, the TIDieR checklist should be used in conjunction with the CONSORT statement (see [www.consort-statement.org](http://www.consort-statement.org)) as an extension of **Item 5 of the CONSORT 2010 Statement.** When a **clinical trial** **protocol** is being reported, the TIDieR checklist should be used in conjunction with the SPIRIT statement as an extension of **Item 11 of the SPIRIT 2013 Statement** (see [www.spirit-statement.org](http://www.spirit-statement.org)). For alternate study designs, TIDieR can be used in conjunction with the appropriate checklist for that study design (see [www.equator-network.org](http://www.equator-network.org)).
