# Supplementary material for: A natural experimental study of improvements along an urban canal: impact on canal usage, physical activity and other wellbeing behaviours
Source: Int J Behav Nutr Phys Act. 2021 Jan 27;18:19. doi: 10.1186/s12966-021-01088-w (PMC7838466; doi:10.1186/s12966-021-01088-w)
Supplement: Supplementary file 5 — Additional file 5. Completed STROBE Checklist. [file 12966_2021_1088_MOESM5_ESM.docx]

**Additional file 5.** STROBE Statement—checklist of items that should be included in reports of observational studies

|  | Item No. | Recommendation | Page  No. | Relevant text from manuscript |
| --- | --- | --- | --- | --- |
| **Title and abstract** | 1 | (*a*) Indicate the study’s design with a commonly used term in the title or the abstract | Title | ‘A natural experimental study’ |
|  |  | (*b*) Provide in the abstract an informative and balanced summary of what was done and what was found |  |  |
| Introduction | | | |  |
| Background/rationale | 2 | Explain the scientific background and rationale for the investigation being reported | 5-7 | Various points made in the Background  e.g. ‘There is a dearth of natural experimental studies on the effect of urban green space interventions on physical activity and these studies have weak designs’ |
| Objectives | 3 | State specific objectives, including any prespecified hypotheses | 7-8 | ‘The aim of this two-year natural experimental study was to evaluate the impact of the canal improvements on canal usage, physical activity and two other wellbeing behaviours (social interactions and taking notice of the environment) in adults. In line with the published study protocol, pre-planned objectives were to examine whether the canal improvements increased the following at 7, 12 and 24 months post-baseline, compared to matched comparison sites where no changes occurred:   1. Total number of people (primary outcome at 12 months); 2. Walking and Vigorous physical activity behaviours (secondary outcomes); 3. Sedentary, Connect and Take Notice behaviours (exploratory outcomes).   A further objective was to explore potential displacement of activity from a separate existing canal path, through process evaluation using intercept surveys and systematic observation.’ |
| Methods | | | |  |
| Study design | 4 | Present key elements of study design early in the paper | 8 | ‘This was a prospective controlled natural experimental study, with one intervention site and two matched comparison sites’ |
| Setting | 5 | Describe the setting, locations, and relevant dates, including periods of recruitment, exposure, follow-up, and data collection | 8, 18 and 22 | ‘The study was set in Greater Manchester, UK: a large metropolitan authority containing ten boroughs.’  ‘We conducted observations at baseline (Nov 2017) and at three follow-ups: 7 months (June 2018), 12 months (Nov 2018) and 24 months (Nov 2019) post-baseline. Observations were conducted during four hour-long observation periods (8-9am, 10-11am, 1-2pm, 3-4pm) on three days (Tuesday, Wednesday, Saturday) i.e. twelve hours of observations for each site at each time point.’  ‘Surveys were conducted at each of the follow-ups (June/ July 2018; Nov/ Dec 2018; Nov/ Dec 2019) at the same time of day as observations, but on different days.’ |
| Participants | 6 | (*a*) *Cohort study*—Give the eligibility criteria, and the sources and methods of selection of participants. Describe methods of follow-up  *Case-control study*—Give the eligibility criteria, and the sources and methods of case ascertainment and control selection. Give the rationale for the choice of cases and controls  *Cross-sectional study*—Give the eligibility criteria, and the sources and methods of selection of participants | 14 and 19 | ‘The target area at the intervention site was the path identified for improvements (‘intervention path’). Observers recorded anyone who entered onto the intervention path, regardless of which direction they were coming from. Target areas at the two comparison sites were defined to resemble the target area at the intervention site, by capturing people who entered the unsurfaced canal side path regardless of which direction they were coming from.’  ‘Surveys were attempted with all English-speaking adult canal users (aged 18+ checked in introduction).' |
|  |  | (*b*) *Cohort study*—For matched studies, give matching criteria and number of exposed and unexposed  *Case-control study*—For matched studies, give matching criteria and the number of controls per case | Pages 9 and 10  Figure 3 and Additional file 1 | ‘We used a recently developed five-step process to identify two matched comparison sites in Greater Manchester (see Figure 3) [30]. The eight variables used for matching were based on several systematic reviews of physical activity environmental correlates in adults [31–36] and older adults [37–39]. In brief, the first step involves identifying the most closely matched neighbourhoods to the index intervention neighbourhood, using spatial data at the Lower Layer Super Output Area (LSOA) level (population density, street connectivity, deprivation, neighbourhood greenness). The next four steps involve searching for the most closely matched comparison sites within the potential matched neighbourhoods identified in step one, using variables at the site level (e.g. footpath, benches, lighting). Steps two and three are conducted using Google Street View to narrow down potential matched comparison sites. Steps four and five involve in-person site audits. Additional file 2 provides further details of this matching process.’ |
| Variables | 7 | Clearly define all outcomes, exposures, predictors, potential confounders, and effect modifiers. Give diagnostic criteria, if applicable | Pages 9, 10 and 19  Figure 3 and Additional file 1 | ‘‘We used a recently developed five-step process to identify two matched comparison sites in Greater Manchester (see Figure 3) [30]. The eight variables used for matching were based on several systematic reviews of physical activity environmental correlates in adults [31–36] and older adults [37–39]. In brief, the first step involves identifying the most closely matched neighbourhoods to the index intervention neighbourhood, using spatial data at the Lower Layer Super Output Area (LSOA) level (population density, street connectivity, deprivation, neighbourhood greenness). The next four steps involve searching for the most closely matched comparison sites within the potential matched neighbourhoods identified in step one, using variables at the site level (e.g. footpath, benches, lighting). Steps two and three are conducted using Google Street View to narrow down potential matched comparison sites. Steps four and five involve in-person site audits. Additional file 2 provides further details of this matching process.’  ‘Observations were carried out regardless of weather conditions; observation periods were coded as high precipitation if the observer recorded an accumulated duration of any precipitation lasting for 50% or more of the observation period (i.e. 30 minutes or more), as recommended by MOHAWk procedures.’ |
| Data sources/ measurement | 8* | For each variable of interest, give sources of data and details of methods of assessment (measurement). Describe comparability of assessment methods if there is more than one group | 18 and 19 | ‘Observations were conducted using MOHAWk (Method for Observing pHysical Activity and Wellbeing): a systematic observation tool for assessing three levels of physical activity (Sedentary, Walking, Vigorous) and two other wellbeing behaviours (Take Notice: taking notice of the environment; Connect: social interactions) in urban spaces [21].’  ‘JB trained all observers using the MOHAWk instruction manual and by practicing observations in the study sites. Inter-rater reliability between each pair of observers for assessing agreement on counts of people, their characteristics and behaviours was ‘good’ or ‘excellent’, analysed using two-way mixed, single measure, consistency intraclass correlation coefficients (ICCs) (< 0.5 = poor; 0.5 – 0.75 = moderate; 0.76 – 0.9 = good; and > 0.9 = excellent [45]).’ |
| Bias | 9 | Describe any efforts to address potential sources of bias | 31 and 32 | ‘This natural experimental study offers a feasible exemplar of how to address several major methodological weaknesses causing high risk of bias in previous studies in this area. Key strengths of the present study include identifying multiple matched comparison sites using important objective variables (e.g. population density); adjusting for key covariates to minimise the risk of confounding using methods recommended by MRC guidance [10]; conducting a sample size calculation; publication of a study protocol with a priori analyses specified and reporting any key deviations; clear reporting of samples and interventions in line with standardised checklists; and a mixed-methods approach. These are substantial improvements on many previous natural experimental studies that have often used poorly matched single comparison groups (rarely matched on objective variables); not controlled for key confounding factors by study design or in the analyses; not attempted sample size calculations; not published study protocols; often relied on single outcome measures (sometimes unvalidated); and are poorly reported [9,11,13].’ |
| Study size | 10 | Explain how the study size was arrived at | 20 | ‘We used an approach suggested by Donner and Klar ([47], p.66) for calculating the sample size for a matched pair design. This was conducted for the primary outcome measure: total number of people per observation period (hour). Due to the absence of studies of canal paths on which to base our estimates, the sample size calculation utilised MOHAWk data from a feasibility study of two UK residential streets [30]. Matching one intervention site and two comparison sites, with twelve observation periods per site, provided 80% power (p = .05, two-tailed test) to detect a difference between 20 (SD = 27) counts per observation period of the total number of people in the comparison group and 80 (SD = 27) in the intervention group. This assumed an intraclass correlation coefficient (ICC) of 0.02.’ |

Continued on next page

| Quantitative variables | 11 | Explain how quantitative variables were handled in the analyses. If applicable, describe which groupings were chosen and why | 20 | ‘The unit of analysis was at the level of the observation period i.e. counts per observation period per site. Analyses were conducted separately for data at 7, 12 and 24 months post-baseline. Analyses were conducted using Stata version 14.1.20. We originally planned to analyse adults and older adults separately, but combined them to increase statistical power.’ |
| --- | --- | --- | --- | --- |
| Statistical methods | 12 | (*a*) Describe all statistical methods, including those used to control for confounding | 20-21 | ‘We estimated the effect of the intervention on the total number of people counted per observation period, compared to the comparison sites, controlling for three covariates (day, time of day and precipitation). Negative binomial regression models were used because they can account for overdispersion [48].  We followed three steps to build a suitable negative binomial regression model. First, using baseline data only (without group allocation), we built a regression model to examine the relationship between the baseline data and covariates. Second, we combined the baseline and follow-up data, and applied the same regression model from step one. Third, we added Intervention Group (intervention or comparison) and Time Point (baseline or follow-up) into the model. The intervention effect was the interaction of Intervention Group and Time Point. This is a form of difference in differences analysis [49]. We obtained model estimates using the menbreg command in Stata version 14.1.20 i.e. multilevel mixed-effects negative binomial regression.’ |
|  |  | (*b*) Describe any methods used to examine subgroups and interactions | N/A | N/A |
|  |  | (*c*) Explain how missing data were addressed | N/A | N/A |
|  |  | (*d*) *Cohort study*—If applicable, explain how loss to follow-up was addressed  *Case-control study*—If applicable, explain how matching of cases and controls was addressed  *Cross-sectional study*—If applicable, describe analytical methods taking account of sampling strategy | N/A | N/A |
|  |  | (*e*) Describe any sensitivity analyses | 21 | ‘To assess for any potential bias due to precipitation when analysing the physical activity and other wellbeing behaviour outcomes, observation periods were removed if precipitation occurred for 50% or more of the observation period (recorded by the observer).’ |
| Results | | | | |
| Participants | 13* | (a) Report numbers of individuals at each stage of study—eg numbers potentially eligible, examined for eligibility, confirmed eligible, included in the study, completing follow-up, and analysed | Tables 3 and 4 | [Tables 3 and 4 reports total counts of individuals at each stage of study in the intervention and comparison groups] |
|  |  | (b) Give reasons for non-participation at each stage | N/A | [We collected data using direct observations of people entering target areas in each of the intervention and comparison sites; therefore, participants were not ‘recruited’ into the study.] |
|  |  | (c) Consider use of a flow diagram | N/A | N/A |
| Descriptive data | 14* | (a) Give characteristics of study participants (eg demographic, clinical, social) and information on exposures and potential confounders | Table 2 | [Table 2 provides details on baseline characteristics of the sample at baseline in intervention and comparison groups] |
|  |  | (b) Indicate number of participants with missing data for each variable of interest | N/A | [We collected data using direct observations of people entering target areas in each of the intervention and comparison sites; therefore, participants were not ‘recruited’ into the study.] |
|  |  | (c) *Cohort study*—Summarise follow-up time (eg, average and total amount) | 18 | ‘We conducted observations at baseline (Nov 2017) and at three follow-ups: 7 months (June 2018), 12 months (Nov 2018) and 24 months (Nov 2019) post-baseline. Observations were conducted during four hour-long observation periods (8-9am, 10-11am, 1-2pm, 3-4pm) on three days (Tuesday, Wednesday, Saturday) i.e. twelve hours of observations for each site at each time point.’ |
| Outcome data | 15* | *Cohort study*—Report numbers of outcome events or summary measures over time | Tables 3 and Table 4 | [Summary statistics reported for overall counts of people and each of the wellbeing behaviours in the intervention and comparison groups at baseline and 12 months in Tables 3 and Table 4] |
|  |  | *Case-control study—*Report numbers in each exposure category, or summary measures of exposure | N/A | N/A |
|  |  | *Cross-sectional study—*Report numbers of outcome events or summary measures | N/A | N/A |
| Main results | 16 | (*a*) Give unadjusted estimates and, if applicable, confounder-adjusted estimates and their precision (eg, 95% confidence interval). Make clear which confounders were adjusted for and why they were included | 23-24 | ‘Compared to the comparison sites, the total number of people observed using the intervention canal path increased at all three follow-ups, controlling for day, time of day and precipitation: 7 months (incidence rate ratio (IRR) 1.67, 95% CI 1.44 – 1.95), 12 months (primary outcome) (IRR 2.10, 95% CI 1.79 – 2.48), and 24 months post-baseline (IRR 2.42, 95% CI 1.80 – 3.24).’  ‘Walking behaviour increased in the intervention site at all follow-ups; Vigorous activity increased at 7 months (p = .009) and 24 months (p = .002), but not at 12 months (p = .96) post-baseline (Table 4); Connect behaviour increased at 12 months (p = .03) and 24 months (p = .006), but not at 7 months (p = .42) post-baseline (Table 4); and Take Notice behaviour increased at 24 months (p = .001), but not at 7 months (p = .07) or 12 months (p = .58) post-baseline (Table 4).’ |
|  |  | (*b*) Report category boundaries when continuous variables were categorized | N/A | N/A |
|  |  | (*c*) If relevant, consider translating estimates of relative risk into absolute risk for a meaningful time period | N/A | N/A |

Continued on next page

| Other analyses | 17 | Report other analyses done—eg analyses of subgroups and interactions, and sensitivity analyses | 24 and Additional file 5 | ‘Removing observation periods with high precipitation did not change the direction or statistical significance of the results for the secondary and exploratory outcomes (see Additional file 5).’ |
| --- | --- | --- | --- | --- |
| Discussion | | | | |
| Key results | 18 | Summarise key results with reference to study objectives | 29-30 | ‘The new walking infrastructure and green space improvements were associated with a statistically significant increase in the number of people using the intervention canal path compared to the comparison sites at all time points (up to 24 months post-baseline). There were also statistically significant increases in walking and vigorous physical activity, social interactions, and people taking notice of the environment at the intervention canal path compared to the comparison sites. The process evaluation suggested that there was displacement of activity from the existing unchanged canal path to the intervention path, but displacement from this particular canal route cannot explain all of the observed increases in use of the intervention path. Several survey participants reported that the intervention encouraged them to use the canal more often per visit or more often across the week.’ |
| Limitations | 19 | Discuss limitations of the study, taking into account sources of potential bias or imprecision. Discuss both direction and magnitude of any potential bias | 32-33 | ‘A process evaluation enabled us to strengthen causal inferences from the observations, including possible displacement effects; a key limitation of previous natural experimental studies in this area is not assessing for possible displacement effects [13]. However, we were unable to precisely estimate the effect of the intervention on overall population-levels of physical activity, as we did not include measures that assessed within-person change over time (e.g. surveys, accelerometers). Nonetheless, population-level measures do suffer from certain methodological challenges (e.g. poor response rates [11]), which is why triangulation of different outcome measures is optimal to strengthen causal inferences.  Statistical power was an issue for some of the wellbeing behaviours due to low counts, which prevented us from fitting appropriate regression models that could adjust for the total number of people. Nonetheless, we powered the study for the primary outcome (i.e. total number of people), for which there was sufficient data; MOHAWk data from this study will be valuable in informing sample size calculations and analytical plans in future studies of environmental interventions.’ |
| Interpretation | 20 | Give a cautious overall interpretation of results considering objectives, limitations, multiplicity of analyses, results from similar studies, and other relevant evidence | 30-31 | ‘There are two key reasons why improving access to this particular part of the canal may have caused increased use of this route. First, the resurfaced footpath and removal of encroaching vegetation provided better and safer access to ‘wild’ and interesting green corridors along the canal, thus providing a more pleasant walking and visual experience compared to the existing canal path. This accords with previous research which suggests that physical activity in natural environments is generally more enjoyable [50], and can encourage people to be active for longer and at higher intensities [51]. It is therefore plausible that the intervention may act as a catalyst to encourage and sustain physical activity.  Second, improving access to this side of the canal increased capacity of the canal route for more people. This may have enabled better segregation between canal users with different journey purposes (e.g. walking, jogging, cycling), thus reducing shared space conflict. For example, some of the intercept survey participants reported that they started using the intervention path to avoid collisions with cyclists on the existing well-surfaced towpath on the other side of the canal. Previous research suggests that segregating pedestrians and cyclists on shared use routes may be an effective way to reduce collisions and conflict [52], which is very relevant to canals paths as they are generally quite narrow but are used by a variety of users. Reduction in conflict between canal users may have contributed to the observed increases in social interactions at the intervention path 12 and 24 months post-baseline, thus further enhancing the physical activity experience due to the additional wellbeing benefits associated with more social contact, such as lower levels of stress and social isolation [53] and increased feelings of safety and trust [57]. In light of the current COVID-19 pandemic, the pertinence of reducing shared space conflict will be heightened because of social distancing rules to reduce virus transmission [54].  Whilst this is the first natural experimental study of an urban canal intervention on physical activity to date, these findings are consistent with a similar recent controlled natural experimental study in the UK. They found that a comparable intervention to improve access to urban woodlands (e.g. installing new footpaths, clearing overgrown vegetation) caused people to visit the woods more often, and increased physical activity levels, social cohesion and connectedness with nature [55]. They also found that the intervention enhanced people’s experience of the woodlands, as shown by improvements in perceived restorativeness measures (e.g. spending time away from a day-to-day routine, fascination). These findings, together with the present study, suggest that interventions which improve access to existing urban green spaces, especially spaces that enable close physical and visual contact with natural features (e.g. trees, shrubs, water), are effective in causing positive wellbeing behaviour change and improving people’s experience of urban green spaces. Importantly, the intervention providers in the present study included funding for 20 years of ongoing maintenance, which will increase the likelihood of providing long-term benefits and reduce negative effects on wellbeing associated with unmanaged green spaces (e.g. fear of crime) [56].’ |
| Generalisability | 21 | Discuss the generalisability (external validity) of the study results | 35 | ‘It is clear that more well-developed theory is needed to improve our understanding of urban green space interventions, particularly how physical and social contexts impact intervention effectiveness (e.g. deprivation, population demographics). A recent systematic review has begun to develop context-sensitive theory of how environmental interventions work to influence physical activity using realist evaluation methods [70]. The authors found that interventions targeting accessibility of urban environments may provide an effective approach for increasing physical activity that is less sensitive to contextual factors [70]; this suggests that the intervention in the present study may be effective in other contexts, such as more deprived areas. However, theoretical models need to incorporate other wellbeing behaviours beyond physical activity, especially social interactions given the role these behaviours can have in supporting physical activity [71]. This mixed methods study contributes robust evidence of intervention effectiveness required to advance theoretical understanding on physical activity and other wellbeing behaviours.’ |
| Other information | |  | | |
| Funding | 22 | Give the source of funding and the role of the funders for the present study and, if applicable, for the original study on which the present article is based | 37 | ‘JB is funded by the Economic and Social Research Council (ESRC) as part of the North-West Social Science Doctoral Training Partnership (ES/P000665/1). DF is supported by the NIHR Biomedical Research Centre in Manchester (IS-BRC-1215-20007). The views expressed are those of the authors and not necessarily those of ESRC, NIHR or the Department of Health. ‘ |

*Give information separately for cases and controls in case-control studies and, if applicable, for exposed and unexposed groups in cohort and cross-sectional studies.

**Note:** An Explanation and Elaboration article discusses each checklist item and gives methodological background and published examples of transparent reporting. The STROBE checklist is best used in conjunction with this article (freely available on the Web sites of PLoS Medicine at http://www.plosmedicine.org/, Annals of Internal Medicine at http://www.annals.org/, and Epidemiology at http://www.epidem.com/). Information on the STROBE Initiative is available at www.strobe-statement.org.
